# HSPA12A unstabilizes CD147 to inhibit lactate export and migration in human renal cell carcinoma

**DOI:** 10.7150/thno.44321

**Published:** 2020-07-09

**Authors:** Xinxu Min, Xiaojin Zhang, Yunfan Li, Xiaofei Cao, Hao Cheng, Yuehua Li, Chuanfu Li, Qiuyue Kong, Qian Mao, Peipei Peng, Yan Ni, Jingjin Li, Yulian Duan, Li Liu, Zhengnian Ding

**Affiliations:** 1 Department of Anesthesiology, Jiangsu Provincial Key Laboratory of Geriatrics, the First Affiliated Hospital with Nanjing Medical University, Nanjing 210029, China;; 2 Department of Geriatrics, First Affiliated Hospital with Nanjing Medical University, Nanjing 210029, China;; 3 Key Laboratory of Targeted Intervention of Cardiovascular Disease, Collaborative Innovation Center for Cardiovascular Disease Translational Medicine, Nanjing Medical University, Nanjing 210029, China;; 4 Department of Surgery, East Tennessee State University, Johnson City, TN 37614.

**Keywords:** renal cell carcinoma, heat shock protein A12A (HSPA12A), migration, cluster of differentiation 147 (CD147), lactate

## Abstract

**Background:** Metastasis accounts for 90% of cancer-associated mortality in patients with renal cell carcinoma (RCC). However, the clinical management of RCC metastasis is challenging. Lactate export is known to play an important role in cancer cell migration. This study investigated the role of heat shock protein A12A (HSPA12A) in RCC migration.

**Methods:** HSPA12A expression was examined in 82 pairs of matched RCC tumors and corresponding normal kidney tissues from patients by immunoblotting and immunofluorescence analyses. The proliferation of RCC cells was analyzed using MTT and EdU incorporation assays. The migration of RCC cells was evaluated by wound healing and Transwell migration assays. Extracellular acidification was examined using Seahorse technology. Protein stability was determined following treatment with protein synthesis inhibitor cycloheximide and proteasome inhibitor MG132. Mass spectrometry, immunoprecipitation, and immunoblotting were employed to examine protein-protein interactions.

**Results:** RCC tumors from patients showed downregulation of HSPA12A, which was associated with advanced tumor node metastasis stage. Intriguingly, overexpression of HSPA12A in RCC cells inhibited migration, whereas HSPA12A knockdown had the opposite effect. Lactate export, glycolysis rate, and CD147 protein abundance were also inhibited by HSPA12A overexpression but promoted by HSPA12A knockdown. An interaction of HSPA12A with HRD1 ubiquitin E3 ligase was detected in RCC cells. Further studies demonstrated that CD147 ubiquitination and proteasomal degradation were promoted by HSPA12A overexpression whereas inhibited by HSPA12A knockdown. Notably, the HSPA12A overexpression-induced inhibition of lactate export and migration were abolished by CD147 overexpression.

**Conclusion:** Human RCC shows downregulation of HSPA12A. Overexpression of HSPA12A in RCC cells unstabilizes CD147 through increasing its ubiquitin-proteasome degradation, thereby inhibits lactate export and glycolysis, and ultimately suppresses RCC cell migration. Our results demonstrate that overexpression of HSPA12A might represent a viable strategy for managing RCC metastasis.

## Introduction

Renal cell carcinoma (RCC) is one of the most frequently diagnosed cancers worldwide, and the incidence rates have been steadily increasing 1-3. More than 10 histological and molecular subtypes of RCC have been identified, among which clear cell RCC (ccRCC) is the most common type and accounting for 80% of all cases 2, 4. The prognosis of RCC is poor because approximately 25% of patients present with metastases at the time of diagnosis, and another 30-35% of patients underwent resection for localized or locally invasive kidney cancer will develop potentially fatal metachronous distant metastases 5, 6. Therefore, a comprehensive understanding of RCC metastasis is urgently needed to develop effective targeted therapies for reducing the risk of recurrence and death from metastatic disease.

Cancer cell invasion and metastasis are complex processes that involve many genetic alterations and subsequent metabolic transitions 7. Cluster of differentiation 147 (CD147) has been shown to play a critical role in metastasis 8, 9. CD147, also known as Basigin or extracellular matrix metalloproteinase inducer (EMMPRIN), is a multifunctional glycoprotein involved in various biological functions including cell proliferation, survival, and invasion, and is expressed at high levels in a variety of human cancers 10-13. The N-terminal domain of CD147 contains three glycosylation sites, which results in its molecular mass ranges from 27 kDa for the non-glycosylated form (NoG-CD147) to 32 kDa for the low-glycosylated (LG-CD147) form, and 40-65 kDa for high-glycosylated CD147 (HG-CD147) according to immunoblot analysis 13, 14. HG-CD147 is the mature and active form for facilitating the translocation of monocarboxylate transporters MCT1 and MCT4 to the membrane 9, 13, 15. CD147 also promotes the expression of MCT1 and MCT4 16, 17. MCT1 facilitates lactate uptake whereas MCT4 favors lactate export to increase the content of lactate and acidification of the tumor microenvironment that leading to cancer cell migration and invasion 9, 18, 19. There is convincing evidence that CD147 increases the efficacy of lactate export through MCT4 thereby removing the feedback inhibitory effect of cellular lactate on glycolytic flux 9, 20. Thus, CD147 inhibition has been proposed as a potential therapeutic anti-cancer approach. However, the regulation of CD147 in cancer cells has not been clearly clarified.

Heat shock protein A12A (HSPA12A) is a novel but atypical member of the HSP70 family 21. *Hspa12a* mRNA is expressed at high levels in the human and murine brains under normal conditions, whereas its expression is decreased in schizophrenia patients 21, 22. We recently showed that HSPA12A mediates a pro-survival pathway against cerebral ischemic injury, and it also promotes high-fat diet-induced non-alcoholic liver disease and obesity 23, 24. Besides its elevated expression levels in the brain, HSPA12A is highly expressed in the kidney 23, 24, suggesting that it might play a role in the maintenance of renal homeostasis. However, the involvement of HSPA12A in renal disorders including renal cancers remains to be investigated.

In this study, we found that RCC tumors from patients showed downregulation of HSPA12A, which was associated with advanced tumor node metastasis (TNM) stage and Fuhrman grade. In loss- and gain-of-function experiments, HSPA12A overexpression inhibited RCC cell migration whereas HSPA12A knockdown had the opposite effect. Molecular studies revealed that HSPA12A decreased CD147 protein stability by promoting ubiquitin-proteasomal degradation, thereby inhibited lactate and glycolysis, and ultimately suppressed RCC cell migration. These findings suggest that HSPA12A is a novel suppressor of RCC migration. Thus, HSPA12A overexpression might represent a viable strategy for preventing metastasis in human RCC.

## Methods

### Reagents

Modified McCoy's 5A medium and MTT [3-(4, 5-Dimethylthiazol-2-yl)-2, 5-diphenyltetrazolium bromide] reagent were from Sigma-Aldrich (St. Louis, MO). Trizol reagent and Lipofectamine 3000 were from Life Technology (Carlsbad, CA). Bovine serum albumin (BSA) was from Roche (Basel, Switzerland). Normal Goat Serum was from Jackson ImmunoResearch (West Grove, PA). RPMI 1640 medium and fetal bovine serum (FBS) were from Biological Industries (Kibbutz Beit Haemek, ISRAEL). High-sig ECL western blotting substrate was from Tanon (Shanghai, China). Protein A-Agarose was from Santa Cruz Biotechnology (Dallas, TX). Cell-Light^TM^ EdU Apollo^®^567 In Vitro Imaging Kit was from RiboBio (Guangzhou, China). Lactate assay kit was from Jiancheng Biotech (Nanjing, China). MG132, cycloheximide (CHX) and SU6656 were from MedChem Express (Monmouth Junction, NJ).

### Human samples

A total of 82 primary RCC tumor samples were collected from patients who had underwent nephrectomy in the First Affiliated Hospital of Nanjing Medical University (Nanjing, China). All patients were not previously received systemic therapy. Tumor stage and grade were determined after nephrectomy according to the 2010 TNM classification system and the Fuhrman grading system 25, 26. In the present study, we included 72 of clear-cell RCCs, 3 of papillary RCCs, and 1 of chromophobe RCCs, 2 of spindle cell carcinoma, and 4 other types of carcinoma. The Ethical Board of First Affiliated Hospital of Nanjing Medical University approved these studies (#2019-SR-489). Patients gave informed consent at the time of recruitment. All the human studies were conducted according to the principles set out in the WMA Declaration of Helsinki and the Department of Health and Human Services Belmont Report.

### Bioinformatics analysis

Using the TCGA (https://www.cancer.gov/tcga) database, we obtained the standardized expression levels of HSPA12A mRNA in Kidney renal clear cell carcinoma and their association with clinical features, including TNM stage, tumor grade, overall survival and disease free survival.

### Recombinant vectors

The adenoviral vector containing 3 Flags-tagged coding region of *Hspa12*a was generated by GeneChem (Shanghai, China) as described in our previous studies 23, 24. The pTT3 plasmids containing* Cd147* expression coding region (pTT3-CD147) were provided by Addgene (#36147, Cambridge, MA) 27.

### Cell cultures and treatments

Human clear cell carcinoma Caki-1 cells and human renal cell adenocarcinoma 786O cells were grown in modified McCoy's 5A medium and in RPMI 1640 medium, respectively. Both media were supplemented with 10% fetal bovine serum, 100 units/ml penicillin, and 100 μg/ml streptomycin. All cell lines were free of mycoplasma contamination. Cells were plated in 60-mm dishes at a density of 3×10^5^ cells/dish or 24-well plate at a density of 1.5×10^4^ cells/well. The cells were passaged when they reached at 80% confluence.

Overexpression of HSPA12A in cells was established by infection with Flag-tagged HSPA12A-expressing recombinant adenovirus (Ad-HSPA12A) or normal control vectors (Ad-NC). To overexpress CD147, cells were transfected with pTT3-CD147 plasmids or empty pTT3 control vectors using Lipofectamine 3000. Knockdown of HSPA12A was achieved by introducing HSPA12A-targeting siRNA (Si-HSPA12A) or the corresponding scramble negative controls (Si-NC) using siRNA-mate (Genepharma, China). The siRNA sequences were shown in **Table S1**. All the measurements were performed 48 h after gene overexpression or knockdown unless indicated elsewhere. In FAK inhibition experiments, cells were treated with SU6656 (2μM) 1 h prior to HSPA12A knockdown.

### Immunoblotting and immunoprecipitation-immunoblotting

Tissues or cells were subjected to cytosolic and nuclear protein preparation using the lysis buffer A and B, respectively (**Table S2**). Equal amount (30 μg) of proteins was used for immunoblotting according to our previous methods 23, 24. To control for lane loading, the membranes were probed with anti-GAPDH antibodies for cytosolic proteins and anti-Lamin A/C antibodies for nuclear proteins. The developed bands were normalized to the NC control and expressed as the relative levels.

For analysing interaction between HSPA12A and CD147 or HRD1 by immunoprecipitation-immunoblotting, Caki-1 or 786O cells were overexpressed with Flag-tagged HSPA12A. After HSPA12A overexpression for 48 h, cells were collected for protein extraction. Aliquots of equal protein content (0.7 mg) were precipitated with anti-Flag antibodies, followed by Western blotting with anti-CD147, anti-HRD1, and anti-HSPA12A antibodies, as described previously 23.

Antibodies used in the experiments are listed in **Table S3**.

### Immunofluorescence staining

Immunofluorescence staining was performed on 4% PFA-fixed cells or frozen tissue sections according to our previous method 23, 24. Briefly, after incubation with the indicated primary antibodies (1 : 100) overnight at 4 ºC, Cy3- or FITC-conjugated secondary antibody was applied to the samples to visualize the staining. Hoechst 33342 reagent was used to counterstain the nuclei. The staining was observed using a fluorescence microscope and quantified using Cellsens Dimention 1.15 software (Olympus, Tokyo, Japan).

### Quantitative real-time PCR

Quantitative real-time PCR was performed as described previously 23, 24. Briefly, total RNA was extracted and an amount of 2 μg of total RNA was used for cDNA synthesis using the oligo (dT) primer. After cDNA synthesis, the expressions of indicated genes were estimated by real-time PCR using the SYBR Green Master (Roche, Indianapolis, IN). The PCR results of *Gapdh* served as internal controls. We took 2-ΔΔCT method in the calculation. The primers used for PCR were listed in **Table S4**.

### Examination of proliferative ability

**MTT assay.** After knockdown or overexpression of HSPA12A for the indicated times, cell viability was determined using an MTT assay as described previously 28.

#### EdU incorporation

Following overexpression of HSPA12A for 46 h, Caki-1 cells were incubated with EdU for another 2 h. Cell proliferation was indicated by EdU incorporation that visualized by the assay kit according to the manufacture's instruction.

### Examination of migratory ability

#### Wound healing assay

Following overexpression or knockdown of HSPA12A for 48 h, cell monolayers that grown in six-well plates were scratched across the plate to create a streak wound with a 10 μl pipette tip. Progression of migration was observed and photographed at 24 h after wounding and expressed as the migratory distance using computerized Image J software (National Institutes of Health, Bethesda, MD). Three fields on each well were randomly examined with a magnification of 100.

#### Transwell migration assay

Cells were plated in the upper chamber of transwell of 24-well plate (1.5×10^4^ cells/well) following overexpression or knockdown of HSPA12A for 48 h. The pore size of insert was 8 micrometer. After further culture for another 48 h, the migratory cells passing through the insert membrane to the lower chamber were fixed by methanol and stained with 1% crystal violet. The migrated cells into lower chamber were quantified in four randomly **s**elected areas at a magnification of 100 of each sample using Cellsens Dimention 1.15 software (Olympus, Tokyo, Japan).

### Examination of lactate content

Following HSPA12A knockdown or overexpression for 48 h, culture medium and cells were collected for lactate content analysis according to the manufacture's instruction. In another set of experiments, CD147 was overexpressed in Ad-HSPA12A cells for 48 h, and the lactate contents in culture medium and cells were examined subsequently. The lactate values were expressed as relative contents to the respective NC controls.

Also, lactate export was indicated by medium pH values to indicate acidification.

### Measurement of Extracellular acidification rates (ECAR)

ECAR was examined using a Seahorse XF^e^24 Extracellular Flux Analyzer (Seahorse Biosciences, USA). Experiments were performed following manufacturer's protocols. ECAR was assessed using Seahorse XF Glycolysis Stress Test Kit. In Brief, 8 × 10^3^ cells per well were seeded into a Seahorse XF^e^24 cell culture micro-plate and incubated overnight. Glucose, the oxidative phosphorylation inhibitor oligomycin, and the glycolytic inhibitor 2-DG were sequentially injected into each well at indicated time points following baseline measurements. ECAR was shown in mpH/min, and data were analyzed by Seahorse XF^e^24 Wave software.

### Mass spectrometry

Human liver carcinoma HepG2 cells with or without HSPA12A overexpression were used for mass spectrometry analysis according to previous study 23. In brief, the anti-HSPA12A immunoprecipitates were separated by SDS-PAGE followed by Coomassie-blue staining, digested in gel with trypsin, and analyzed by liquid chromatography-tandem mass spectrometry. Peptides were dissolved in solvent A (2% FA in 3% ACN) and loaded directly onto a reversed-phase Trap column (Chrom XP C18-CL-3m 120A, Eksigent). Peptide separation was performed using a reversed-phase analytical column (3C18-CL-120, 3μm, 120A, Eksigent). Eluting peptides from the column were analyzed using an AB Sciex 5600+ TripleTOF™ system. MS/MS data were processed using ProteinPilotTM Software 4.5 (AB Sciex). Tandem mass spectra were searched against UniProt_Homo sapiens (160,566 sequences, released on April 9. 2016) database concatenated with reverse decoy database. Trypsin/P was specified as cleavage enzyme allowing up to 3 missing cleavages, 4 modifications per peptide and 5 charges.

### Flow cytometry

Cells were collected and washed with PBS. After washing, the cells were stained with anti-human CD147 APC for 30 min on ice and in dark. The fluorescence intensity was analyzed by FACSCalibur (Becton Dickinson). Samples without antibody incubation or with anti-mouse IgG1 K isotype control APC served as controls. All of the data were analyzed with the FlowJo Software (Becton Dickinson).

### Statistical analysis

Data represent as mean ± standard deviation (SD). Groups were compared using Student's two-tailed unpaired *t-*test, Student's two-tailed paired *t-*test, one-way ANOVA or two-way ANOVA followed by Bonferroni's test as a post-hoc test. A *P* value of *<* 0.05 was considered as significant.

## Results

### HSPA12A expression is downregulated in human RCC

**Figure 1:**
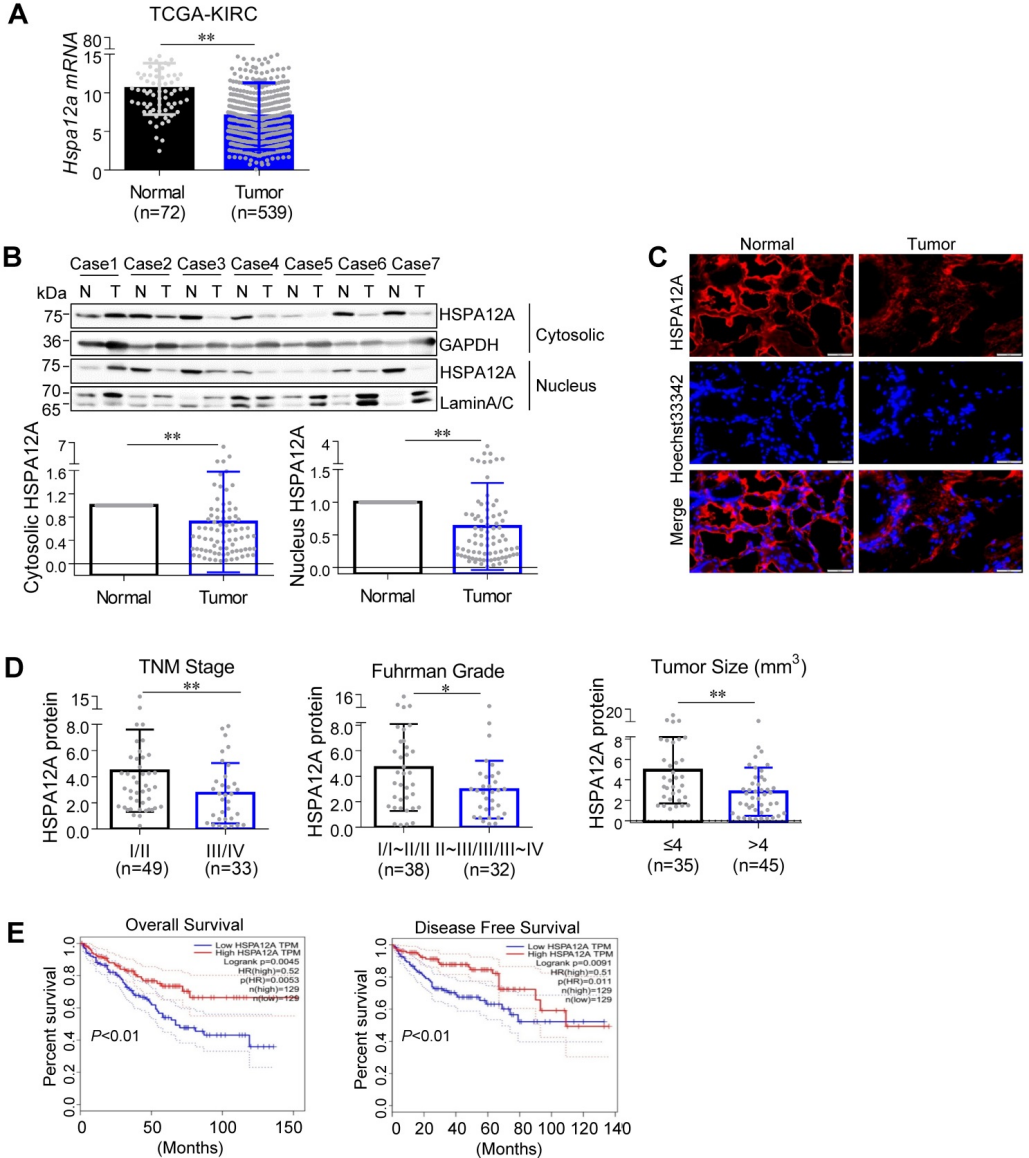
** Downregulated HSPA12A was associated with the poor prognosis of RCC patients. (A)** *Hspa12a* mRNA levels were obtained from TCGA_KIRC database. Sample numbers were indicated in figures. TCGA-KIRC, the Cancer Genome Atlas database for Kidney Renal Clear Cell Carcinoma. **(B)** Human RCC tumors (T) and the matched non-tumor kidney tissues (N) were collected from surgical patients. Cytosolic and nuclear protein extracts were prepared for immunoblotting. n = 82/group. **(C)** Immunofluorescence staining of HSPA12A was performed on frozen sections of RCC tumors and the matched non-tumor kidney tissues of patients. Hoechst 33342 was used to counterstain the nuclei. Scale bar = 50 µm; n = 3 human subjects/group. **(D)** Reduced HSPA12A protein expression was associated with advanced TNM stage, Fuhrman grade and tumor size of human RCC. Sample numbers were indicated in figures. **(E)** Kaplan-Meier curves indicated the reduced HSPA12A mRNA levels were associated with shortened survival of KIRC patients in TCGA cohorts from GEPIA website. n = 129 human subjects/group. * P < 0.05, ** P < 0.01.

To address the role of HSPA12A in RCC pathogenesis, we first analyzed *Hspa12a* mRNA expression by mining The Cancer Genome Atlas (TCGA) database for Kidney Renal Clear Cell Carcinoma (KIRC). The results showed that *Hspa12a* mRNA expression was 33.8% lower in human RCC tissues than in normal controls (**Figure 1A**).

Next, we collected 82 pairs of matched RCC tumors and corresponding normal kidney tissues from human patients and measured HSPA12A protein expression, which was downregulated by 28.3% in cytosolic and 37.3% in pellet fractions of RCC tumors compared with their normal counterparts (**Figure 1B**). Immunofluorescence analysis confirmed the downregulation of HSPA12A in RCC tumors (**Figure 1C**).

### HSPA12A downregulation is associated with unfavorable prognosis in RCC patients

To determine the clinical significance of HSPA12A downregulation in RCC progression, we analyzed the association between HSPA12A protein expression and various clinicopathological variables in our cohort of 82 RCC patients. Reduced HSPA12A expression was significantly associated with advanced TNM stage, high Fuhrman grade, and large tumor size (**Figure 1D**). Consistent with our results, analysis of TCGA cohorts from the UALCAN website (http://ualcan.path.uab.edu) showed an association between downregulated *Hspa12a* mRNA levels and advanced TNM stage and tumor grade in RCC patients 29 (**Figure S1A-B**). Furthermore, the analysis of the Kaplan-Meier plotter from the GEPIA database (http://gepia.cancer-pku.cn) indicates a significant correlation of *Hspa12a* mRNA downregulation with poor overall survival and disease-free survival in RCC patients 30 (**Figure 1E**). Taken together, our findings suggest that HSPA12A downregulation is associated with poor outcomes in RCC patients.

### Overexpression of HSPA12A reduces migration but not the proliferation of RCC cells

The association of HSPA12A downregulation with poor outcomes in RCC patients led us to investigate the potential effect of HSPA12A on RCC cell growth and migration. To this end, HSPA12A was overexpressed in two different human RCC cell lines (Caki-1 and 786O) by infection with Flag-tagged HSPA12A-expressing recombinant adenovirus (Ad-HSPA12A) or normal vector control (Ad-NC). Knockdown of HSPA12A was achieved by introducing HSPA12A-targeting siRNA (Si-HSPA12A) or the corresponding scramble control (Si-NC). Neither HSPA12A overexpression nor its knockdown affected the viability of Caki-1 and 786O cells compared with their respective controls (**Figure S2A**). Also, cell proliferation was not changed by HSPA12A overexpression in Caki-1 cells, as indicated by EdU incorporation (**Figure S2B**). Consistent with these results, the mRNA and protein expression of genes associated with cell survival and proliferation, such as Bax, Bcl-2, C-Myc, and Cyclin D1, showed no changes in response to either HSPA12A overexpression or HSPA12A knockdown in Caki-1 cells (**Figure S3A-B**).

**Figure 2:**
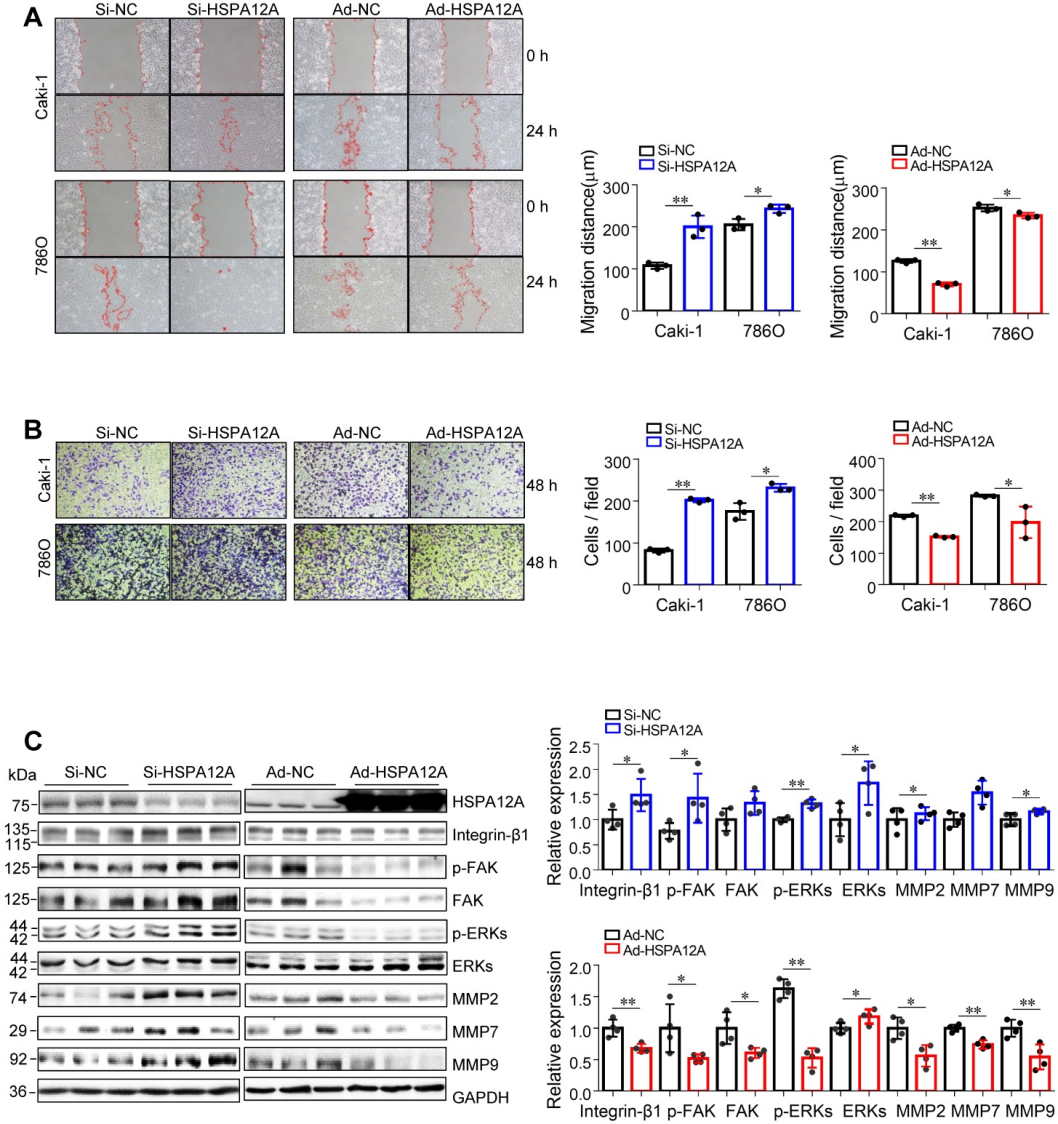
** HSPA12A negatively regulated migration of RCC cells. (A)** The wound was made in layers of Caki-1 and 786O cells after overexpression or knockdown of HSPA12A for 48 h. Following recovery for 24 h, the wound healing was examined and expressed as migration distance (µm). n = 3/group. **(B)** The extent of cell migration was assessed in Caki-1 and 786O cells after overexpression or knockdown of HSPA12A for 48 h by Transwell migration assay. The migrated cells stained with crystal violet were observed 48 h later. n = 3/group. **(C)** Immunoblotting was performed in Caki-1 cells following overexpression or knockdown of HSPA12A for 48 h. n = 4/group. * P < 0.05, ** P < 0.01. Si-NC, cells transfected with Scramble siRNA; Si-HSPA12A, cells transfected with HSPA12A-targeted siRNA; Ad-NC, cells infected with empty adenovirus; Ad-HSPA12A, cells infected with HSPA12A-adenovirus.

We then examined whether HSPA12A affects the migratory ability of RCC cells. The wound closure assays showed that knockdown of HSPA12A in both Caki-1 and 786O cells significantly increased cell migration into the wounds compared with that in Si-NC controls (**Figure 2A**). This was also supported by the Transwell migration assay results, which demonstrated a greater number of Caki-1 and 786O knockdown cells passing through the insert membranes than Si-NC cells (**Figure 2B**). By striking contrast, HSPA12A overexpression markedly suppressed cell migration for wound closure and the ability to pass through the Transwell insert membranes in both Caki-1 and 786O cells (**Figure 2A-B**). The integrin/FAK/ERKs signaling-mediated matrix metalloprotease (MMP) expression has been shown to be critical for cancer metastasis 31-34. Western blot analysis indicated that HSPA12A knockdown promoted, whereas HSPA12A overexpression inhibited integrin-β1, MMP2, and MMP9 expression in Caki-1 cells compared with their respective controls (**Figure 2C**). Also, FAK and ERKs phosphorylation were promoted by HSPA12A knockdown whereas inhibited by HSPA12A overexpression in Caki-1 cells. Similarly, in 786O cells, overexpression of HSPA12A reduced expression of integrin-β1 and MMP2 and phosphorylation of FAK and ERKs compared to their respective NC controls (**Figure S4**). Collectively, the data suggest that HSPA12A negatively regulates RCC cell migration.

To further determine the role of FAK-related signaling in the migratory regulation of HSPA12A, we treated Si-HSPA12A and Si-NC Caki-1 cells with SU6656, a widely used FAK inhibitor that suppresses FAK phosphorylation 35, 36. Both wound healing and Transwell migration assays demonstrated that SU6656 abolished the HSPA12A knockdown-induced increase of migration (**Figure S5A-C**). Moreover, no difference in cell migration between Si-NC and Si-HSPA12A groups was observed in the presence of SU6656. The data suggest that FAK signaling is involved in the HSPA12A knockdown-induced promotion of RCC cell migration.

### Overexpression of HSPA12A inhibits lactate export and glycolysis in RCC cells

**Figure 3:**
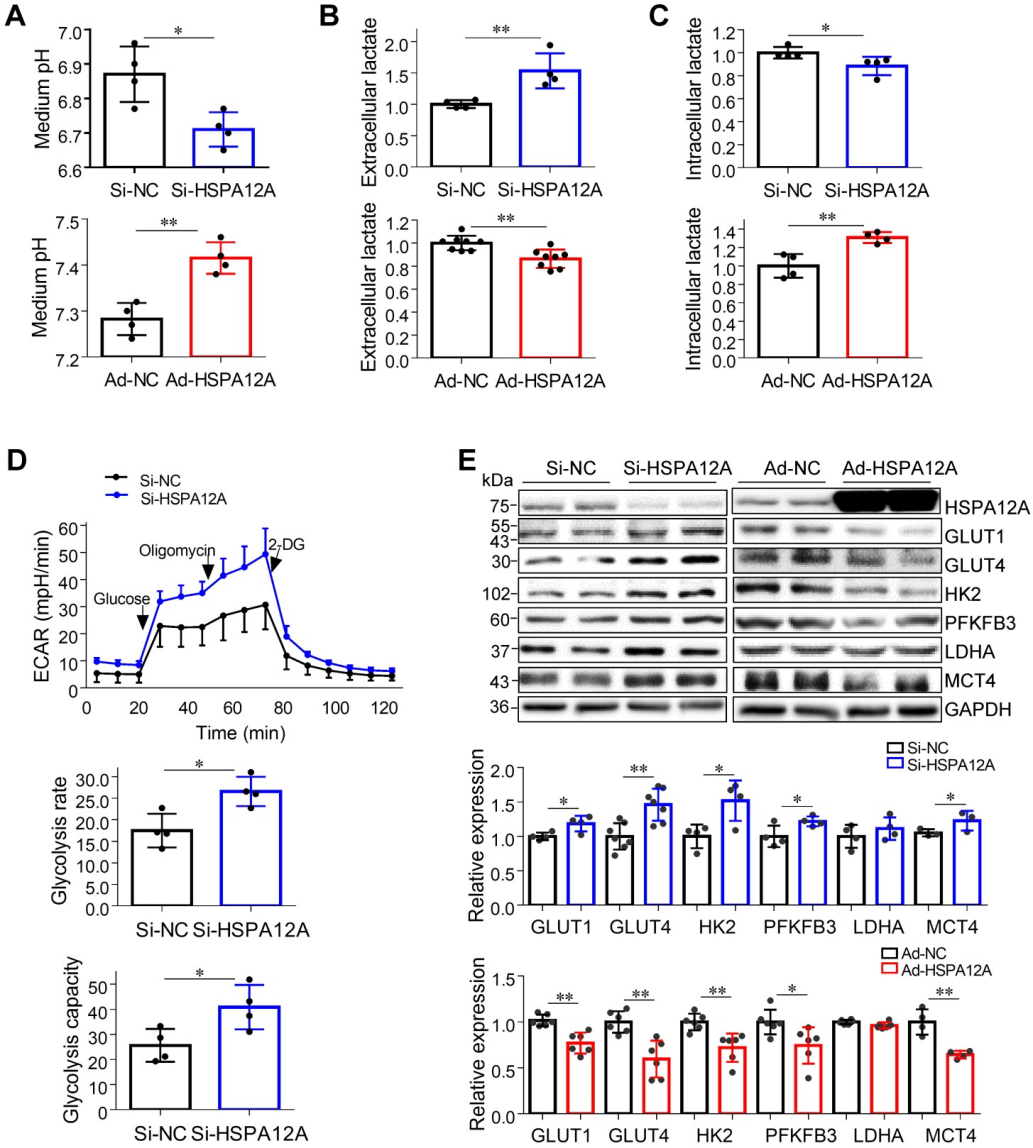
** HSPA12A negatively regulated lactate output and glycolysis of Caki-1 cells. (A)** Medium pH values were measured in Caki-1 cultures after knockdown or overexpression of HSPA12A for 48 h. n = 4/group. **(B)** Extracellular (medium) lactate contents were examined in Caki-1 cultures after knockdown or overexpression of HSPA12A for 48 h. n = 4-8/group. **(C)** Intracellular lactate contents were examined in Caki-1 cells after knockdown or overexpression of HSPA12A for 48 h. n = 4/group. **(D)** Extracellular acidification rate of Caki-1 cells was detected after HSPA12A knockdown for 48 h using the Seahorse extracellular flux analyzer. Glycolysis rate represents extracellular acidification rate after glucose treatment. Glycolysis capacity represents extracellular acidification rate after oligomycin treatment. n = 4/group. **(E)** Immunoblotting was performed in Caki-1 cells following knockdown or overexpression of HSPA12A for 48 h. n = 4-7/group. * *P* < 0.05, ** *P* < 0.01. Si-NC, cells transfected with Scramble siRNA; Si-HSPA12A, cells transfected with HSPA12A-targeted siRNA; Ad-NC, cells infected with empty adenovirus; Ad-HSPA12A, cells infected with HSPA12A-adenovirus.

**Figure 4:**
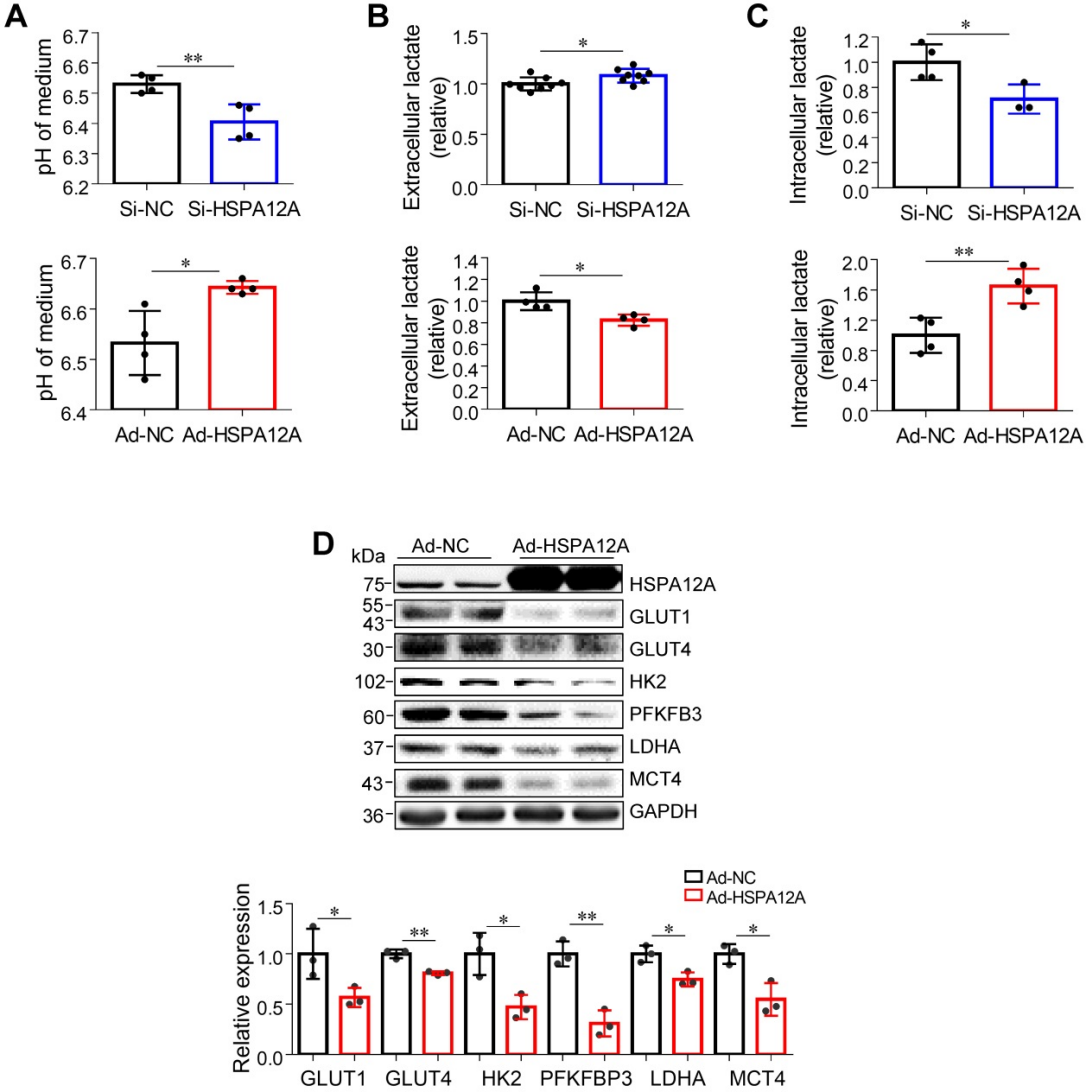
** Downregulation of HSPA12A promoted lactate export and glycolysis of 786O cells. (A)** pH values in medium of 786O cells were measured following knockdown or overexpression of HSPA12A for 48 h. n = 4/group. **(B)** Extracellular (medium) lactate contents were examined in 786O cultures after knockdown or overexpression of HSPA12A for 48 h. n = 8/group (upper panel) and n = 4/group (down panel). **(C)** Intracellular lactate contents were examined in 786O cells after knockdown or overexpression of HSPA12A for 48 h. n = 4/group except for n = 3 in Si-HSPA12A group. **(D)** Immunoblotting was performed in 786O cells following overexpression of HSPA12A for 48 h. n = 3/group. * P < 0.05, ** P < 0.01. Si-NC, cells transfected with Scramble siRNA; Si-HSPA12A, cells transfected with HSPA12A-targeted siRNA; Ad-NC, cells infected with empty adenovirus; Ad-HSPA12A, cells infected with HSPA12A-adenovirus.

Lactate, once considered a waste product of glycolysis, has emerged as a critical regulator of cancer development, maintenance, and metastasis 37-40. We found that the extracellular lactate content and medium acidification of Caki-1 cells were increased by HSPA12A knockdown compared with those of Si-NC controls (**Figure 3A-B**). By contrast, HSPA12A overexpression decreased extracellular lactate content and medium acidification compared with those in Ad-NC controls (**Figure 3A-B**). To exclude the possibility that this change of extracellular lactate was due to the augmented lactate generation and therefore increased lactate output, we measured the intracellular lactate content. The results showed that the intracellular lactate contents were decreased by HSPA12A knockdown but increased by HSPA12A overexpression in Caki-1 cells compared with the controls (**Figure 3C**). Similar effects of HSPA12A on medium acidification, lactate export, and intracellular lactate content were observed in 786O cells (**Figure 4A-C**). Our findings were supported by previous reports that intracellular lactate accumulation serves as a negative feedback signal for glycolysis 9, 20.

To further analyze the effect of HSPA12A on RCC glycolysis, extracellular acidification rates (ECAR) were examined using Seahorse technology. A significant increase in glycolysis rate and capacity was detected in HSPA12A knockdown Caki-1 cells compared to the controls (**Figure 3D**). Consistent with these observations, knockdown of HSPA12A in Caki-1 cells upregulated the expression of genes regulating glucose uptake (GLUT1 and GLUT4), glycolysis (HK2 and PFKFB3) and lactate export (MCT4) (**Figure 3E**). By contrast, overexpression of HSPA12A in Caki-1 cells downregulated the expression of GLUT1, GLUT4, HK2, PFKFB3 and MCT4 in Caki-1 cells compared to their Ad-NC controls. Similarly, the expression of GLUT1, GLUT4, HK2, PFKFB3, LDHA, and MCT4 were downregulated in 786O cells following HSPA12A overexpression (**Figure 4D**). These data suggest that HSPA12A negatively regulates lactate export and glycolysis of RCC cells.

### Overexpression of HSPA12A decreases CD147 protein abundance, maturation, and membrane localization

**Figure 5:**
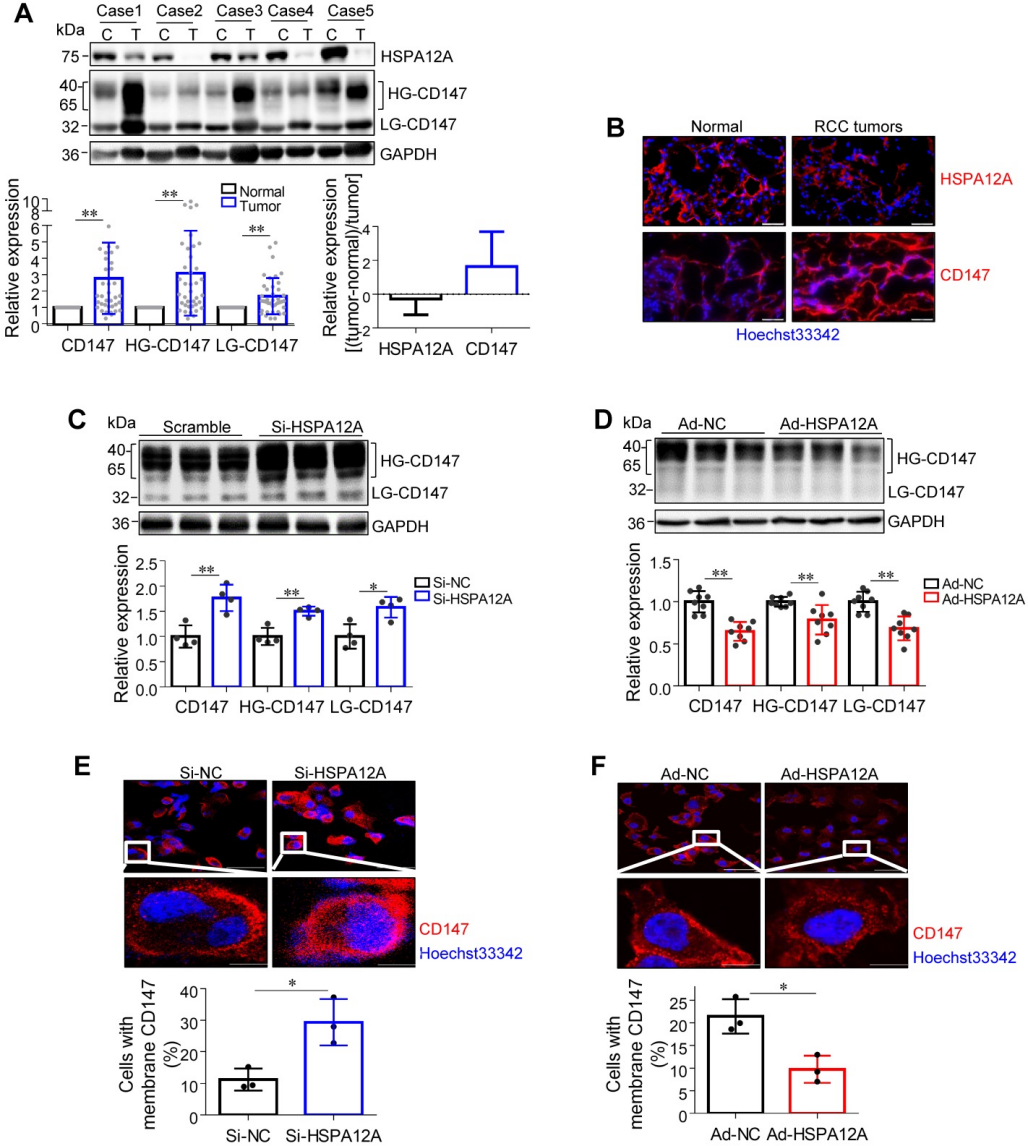
** HSPA12A negatively regulated CD147 protein abundance, maturation and membrane localization. (A)** Human RCC tumors and the matched non-tumor kidney tissues were collected for immunoblotting. n = 40/group. **(B)** Human RCC tumors and the matched non-tumor kidney tissues were prepared for immunofluorescence staining of CD147 and HSPA12A. Hoechst 33342 was used to counterstain the nuclei. Scale bar = 50 µm. n = 3 human subjects/group. **(C,D)** Immunoblotting was performed in Caki-1 cells following knockdown (C) or overexpression (D) of HSPA12A for 48 h. n = 4-8/group. **(E,F)** Immunofluorescence staining against CD147 was performed in Caki-1 cells following knockdown (E) or overexpression (F) of HSPA12A for 48 h. Hoechst 33342 was used to counterstain the nuclei. Scale bar = 50 µm. n = 3/group. * *P* < 0.05, ** P < 0.01. Si-NC, cells transfected with Scramble siRNA; Si-HSPA12A, cells transfected with HSPA12A-targeted siRNA; Ad-NC, cells infected with empty adenovirus; Ad-HSPA12A, cells infected with HSPA12A-adenovirus; HG-CD147, high-glycosylation CD147; LG-CD147, low-glycosylation CD147.

The transmembrane glycoprotein CD147 has been shown to promote migration and glycolysis of cancer cells via MCT4-mediated lactate export 9, 15, 27, 41-43. Based on our previous unbiased screen with mass-spectrometric analysis, showing an interaction between HSPA12A and CD147 in human hepatocellular carcinoma cells (**Figure S6**), we hypothesized that HSPA12A might play a role in the regulation of CD147 during RCC migration. Nonetheless, CD147 protein was not detected in the HSPA12A immunocomplexes of Caki-1 and 786O cells (**Figure S7A-B**), suggesting the lack of direct interaction between HSPA12A and CD147 in RCC cells. However, both immunoblotting and immunostaining analyses revealed increased CD147 protein expression accompanied by reduced HSPA12A expression in human RCC tumors compared to their normal counterparts (**Figure 5A-B**), indicating that HSPA12A might negatively regulate CD147 expression. Indeed, CD147 protein levels were increased by HSPA12A knockdown but decreased by HSPA12A overexpression in Caki-1 cells as indicated by both immunoblotting and immunostaining analyses (**Figure 5C-D**). Reduced CD147 expression was also observed in HSPA12A-overexpressing 786O cells compared to NC controls (**Figure S8**). The effects of HSPA12A on CD147 expression was also confirmed by flow cytometry (**Figure S9**).

High glycosylation is an indicator of CD147 maturation for its membrane localization 13. Interestingly, HG-CD147 levels were increased in human RCC tumors accompanied by reduced HSPA12A expression (**Figure 5A**). Moreover, HSPA12A knockdown increased, whereas HSPA12A overexpression decreased HG-CD147 content in Caki-1 cells (**Figure 5C-D**). HSPA12A knockdown also substantially increased the membrane localization of CD147 in Caki-1 cells, whereas HSPA12A overexpression markedly decreased CD147 membrane localization (**Figure 5E-F**). Besides, a decreased HG-CD147 protein content was observed in 786O cells following HSPA12A overexpression (**Figure S8**). These findings collectively indicate that HSPA12A negatively regulates CD147 protein abundance, maturation, and membrane localization in RCC cells.

Given that CD147 promotes lactate export by promoting MCT4 stability, cell surface expression, and function 15, 42, 43, we analyzed the effect of HSPA12A on MCT4 expression. We found that human RCC tumors with lower HSPA12A expression showed significantly higher MCT4 protein expression than their corresponding non-tumor counterparts (**Figure S10A-B**). This observation was confirmed by *in vitro* experiments showing that HSPA12A knockdown increased, whereas HSPA12A overexpression decreased MCT4 protein expression in both Caki-1 and 786O cells compared with control cells (**Figure 3E** and** 4D**). However, neither HSPA12A knockdown nor HSPA12A overexpression affected *Mct4* mRNA levels in Caki-1 cells (**Figure S11**), indicating that HSPA12A may regulate MCT4 at post-transcriptional levels.

### Overexpression of HSPA12A reduces CD147 to mediate anti-migration effects

**Figure 6:**
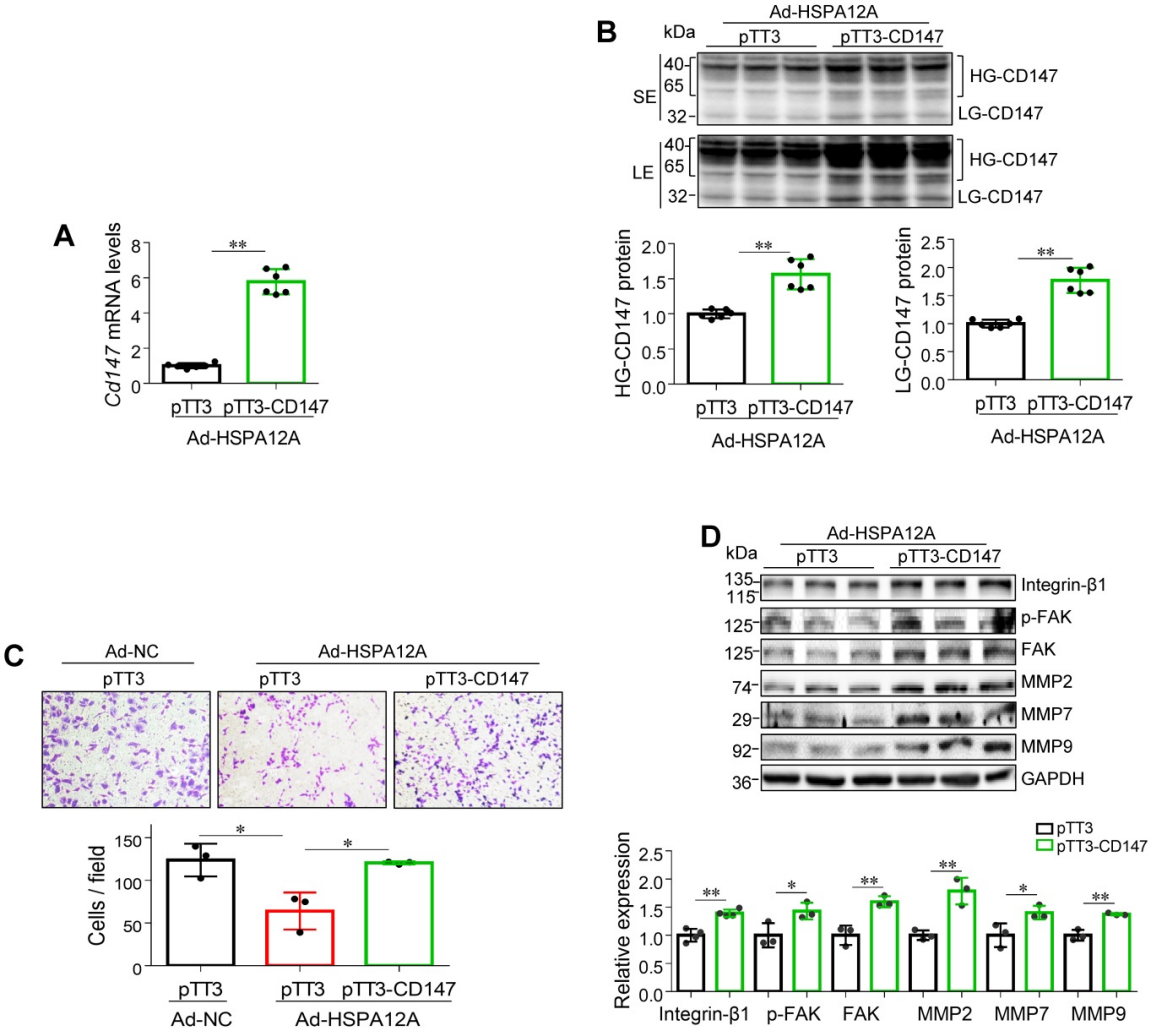
** Overexpression of CD147 reversed the HSPA12A-induced inhibition of RCC cell migration.** Twenty-four hours after HSPA12A overexpression, Caki-1 cells (Ad-HSPA12A) were transfected with pTT3-CD147 plasmids to overexpress CD147 or transfected with pTT3 control plasmids. The following experiments were performed 48 h after plasmid transfection. **(A)** *Cd147* mRNA levels were examined by real-time PCR. n = 6/group. **(B)** CD147 protein levels were examined by immunoblotting. n = 6/group. SE, short exposure; LE, long exposure. **(C)** The extent of cell migration was assessed by Transwell migration assay. n = 3/group. **(D)** Immunoblotting against the indicated proteins was performed. n = 3-4/group. * P < 0.05, ** P < 0.01. Ad-NC, cells infected with empty adenovirus; Ad-HSPA12A, cells infected with HSPA12A-adenovirus; HG-CD147, high-glycosylation CD147; LG-CD147, low-glycosylation CD147; pTT3, empty pTT3 plasmid; pTT3-CD147, plasmid expressing CD147.

To determine whether the anti-migratory activity of HSPA12A overexpression is mediated by decreasing CD147, we overexpressed CD147 in HSPA12A overexpressing-Caki-1 cells (Ad-HSPA12A) by transfection with pTT3-CD147 plasmid or empty pTT3 vector control (**Figure 6A-B**). Notably, overexpression of CD147 reversed the HSPA12A-induced inhibition of cell migration in the Transwell migration assay (**Figure 6C**). Consistent with this observation, overexpression of CD147 in Ad-HSPA12A Caki-1 cells upregulated integrin-β1, MMP2, MMP7, and MMP9 protein expression and increased FAK phosphorylation compared to pTT3 controls (**Figure 6D**). Similar results were observed in 786O cells showing that overexpression of CD147 reversed the HSPA12A-induced inhibition of cell migration in the Transwell migration assay (**Figure S12**) and also increased FAK phosphorylation compared to pTT3 controls (**Figure S13**). These data indicate that HSPA12A inhibits RCC cell migration by reducing CD147.

### Overexpression of HSPA12A reduces CD147 to mediate anti-lactate export and anti-glycolysis effects

**Figure 7:**
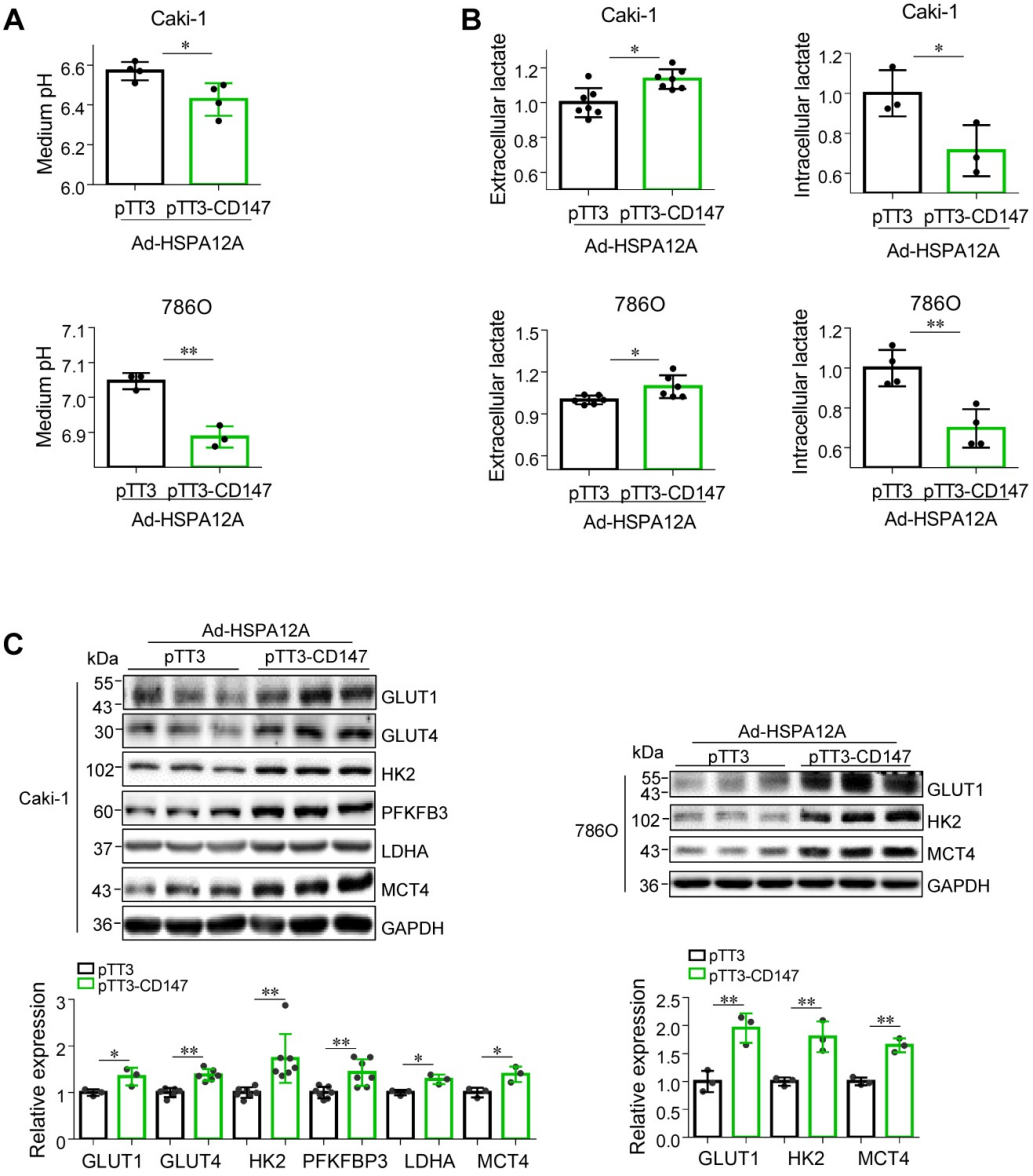
** Overexpression of CD147 diminished the HSPA12A-induced inhibition of lactate output and glycolysis.** Twenty-four hours after HSPA12A overexpression, Caki-1 and 786O cells were transfected with pTT3-CD147 plasmids to overexpress CD147 or transfected with pTT3 control plasmids. The following experiments were performed 48 h after plasmid transfection. **(A)** Medium pH values were measured. n = 3-4/group. **(B)** Lactate contents in both extracellular (medium) and intracellular fractions were examined. n = 3-7/group. **(C)** Immunoblotting against the indicated proteins was performed. n = 3-7/group. * P < 0.05, ** *P <* 0.01. Ad-HSPA12A, cells infected with HSPA12A-adenovirus; pTT3, empty pTT3 plasmid; pTT3-CD147, plasmid expressing CD147.

We next investigated whether the HSPA12A-induced inhibition of lactate export and glycolysis were mediated by the reduction of CD147. We noted that overexpression of CD147 in Ad-HSPA12A Caki-1 and 786O cells increased medium acidification compared with control (pTT3) cells (**Figures 7A**). Consistently, lactate content was increased extracellularly (culture medium) and decreased intracellularly in Ad-HSPA12A Caki-1 and 786O cells following CD147 overexpression (**Figures 7B**). Also, overexpression of CD147 in Ad-HSPA12A cells upregulated protein expression of the genes involved in glucose uptake (GLUT1 and GLUT4), glycolysis (HK2, PFKFB3 and LDHA), and lactate export (MCT4) (**Figure 7C**), compared to their pTT3 controls. These results indicate that HSPA12A inhibits lactate export and glycolysis by reducing CD147 protein expression.

### HSPA12A reduces CD147 protein stability via ubiquitin-proteasome degradation

**Figure 8:**
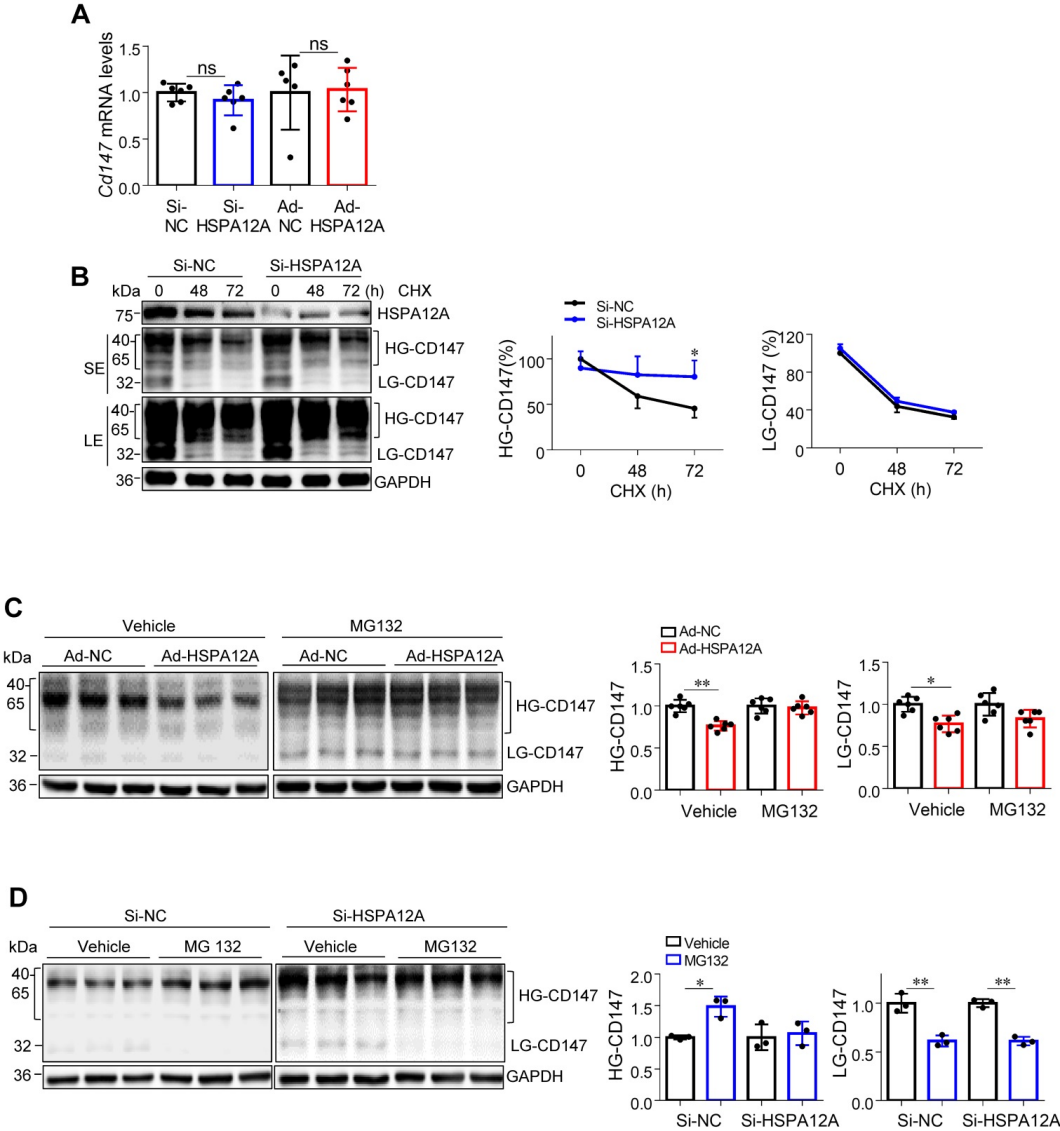
** HSPA12A negatively regulated CD147 stability through proteasomal degradation. (A)** Levels of *Cd147* mRNA were examined in Caki-1 cells following HSPA12A knockdown or overexpression for 48 h. n = 5-6/group. ns, no significance. **(B)** Twenty-four hours after HSPA12A knockdown, Caki-1 cells were treated with cycloheximide (CHX, 150 µg/ml) for the indicated durations. CD147 protein abundance was examined by immunoblotting and expressed as the percentage of the contents at 0 h. n = 3/group. * *P <* 0.05 vs. the time matched Si-NC group. SE, short exposure; LE, long exposure. **(C)** Forty-eight hours after HSPA12A overexpression, Caki-1 cells were treated with MG132 (20 µM) for 8 h. CD147 protein abundance was examined by immunoblotting. n = 6/group. **(D)** Forty-eight hours after HSPA12A knockdown, Caki-1 cells were treated with MG132 (20 µM) for 8 h. CD147 protein abundance was examined by immunoblotting. n = 3/group. * P < 0.05. ** P < 0.01. Si-NC, cells transfected with Scramble siRNA; Si-HSPA12A, cells transfected with HSPA12A-targeted siRNA; Ad-NC, cells infected with empty adenovirus; Ad-HSPA12A, cells infected with HSPA12A-adenovirus; HG-CD147, high-glycosylation CD147; LG-CD147, low-glycosylation CD147.

Next, we explored the potential mechanisms underlying the HSPA12A-mediated regulation of CD147 protein expression. Unexpectedly, neither HSPA12A knockdown nor HSPA12A overexpression had an effect on *Cd147* mRNA levels in Caki-1 and 786O cells that were comparable to the respective NC controls (**Figures 8A** and** S14**). Analysis of the TCGA database for KIRC showed comparable* Cd147* mRNA levels between RCC tumors and their normal controls (**Figure S15**), supporting our observations and suggesting that HSPA12A may regulate CD147 protein stability. To test this hypothesis, we treated Si-HSPA12A Caki-1 cells with cycloheximide (CHX) to block protein translation for 48 and 72 h. Notably, the remaining HG-CD147 protein content, which was expressed as the percentage over NC level at 0 h, was significantly higher in Si-HSPA12A Caki-1 cells than in Si-NC cells after treatment with CHX up to 72 h (**Figure 8B**). Similar results were detected in 786O cells following CHX treatment (**Figure S16**). These data indicate that knockdown of HSPA12A increased CD147 protein stability.

The proteasome is an important system for protein degradation 42. To investigate whether HSPA12A regulates CD147 protein stability by modulating its proteasomal degradation, we examined the effects of the proteasome inhibitor MG132 on HSPA12A-induced changes in CD147 stability in both Caki-1 and 786O cells. The results showed that the HSPA12A overexpression-induced reduction of CD147 was abrogated in the presence of MG132 (**Figures 8C** and** S17A**). Furthermore, CD147 protein level was increased by MG132 in Si-NC control cells, whereas it was comparable between MG132 and vehicle groups in HSPA12A knockdown Caki-1 and 786O cells (**Figures 8D** and** S17B**). The analysis of flow cytometry confirmed that the HSPA12A-induced decrease of CD147 in Caki-1 cells was abolished in the presence of MG132 (**Figure S9**). Taken together, these results suggest that the HSPA12A-induced negative regulation of CD147 protein stability is proteasome-dependent.

**Figure 9:**
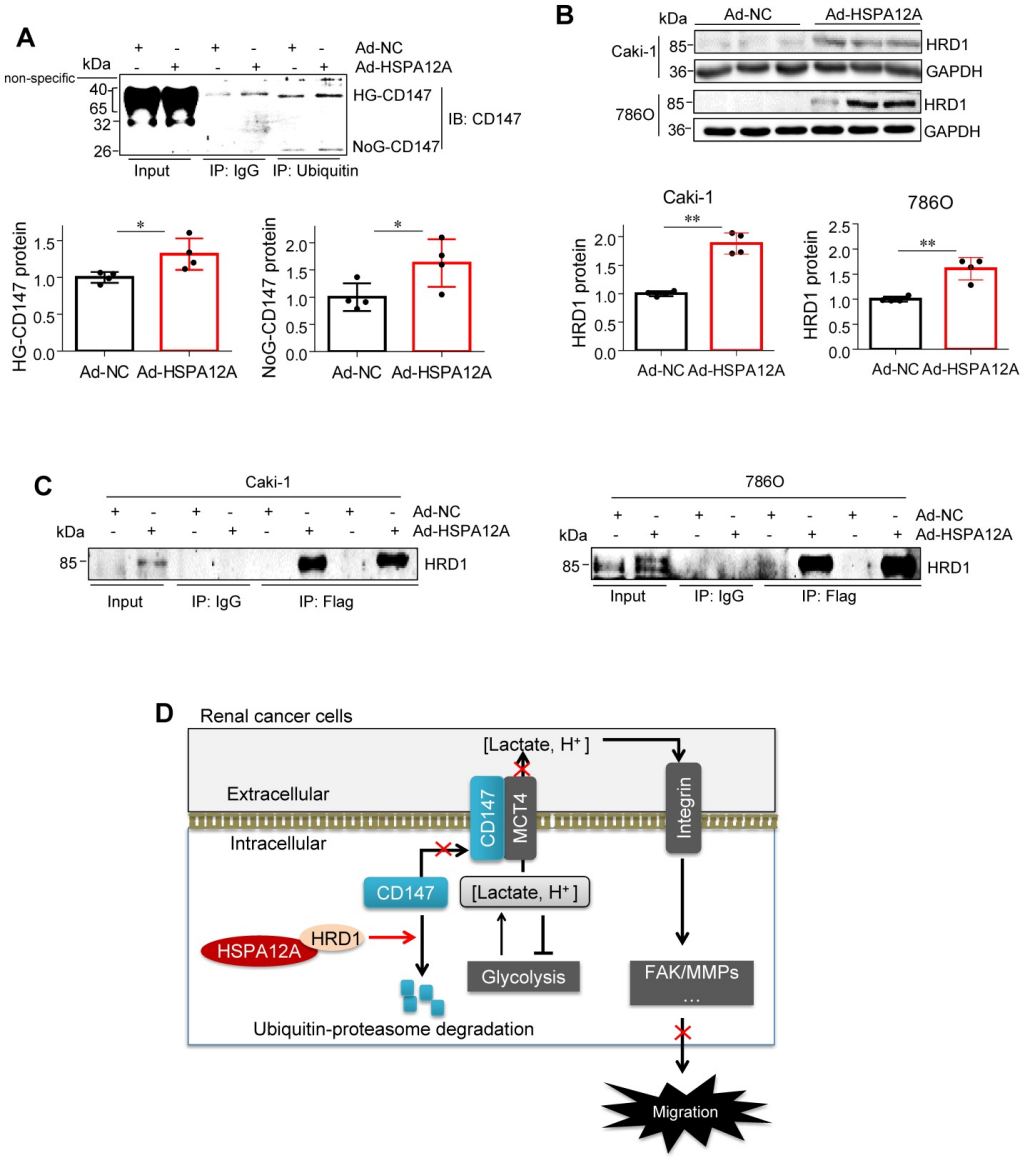
** HSPA12A interacted with ubiquitin E3 ligase HRD1 and increased CD147 ubiquitination. (A)** Immunoprecipitation with ubiquitin was performed in Caki-1 cells following overexpression of HSPA12A for 48 h. Immunoblotting for CD147 was performed in ubiquitin immunoprecipitates. Protein extracts without immunoprecipitation (input) served as positive controls, and immunoprecipitates from IgG incubation served as negative controls. n = 4/group. **(B)** Immunoblotting was performed in Caki-1 and 786O cells following overexpression of HSPA12A for 48 h. n = 4/group. **(C)** Immunoprecipitation with Flag-tagged HSPA12A was performed in Caki-1 and 786O cells following overexpression of HSPA12A for 48 h. Immunoblotting for HRD1 was performed in immunoprecipitates. Protein extracts without immunoprecipitation (input) served as positive controls, and immunoprecipitates from IgG incubation served as negative controls. n = 3/group. **(D)** Mechanistic scheme. HSPA12A interacts with E3 ubiquitin ligase HRD1. Overexpression of HSPA12A in RCC cells unstabilizes CD147 through increasing ubiquitin-proteasome degradation, thereby inhibits lactate export and glycolysis, and ultimately suppresses RCC cell migration. * P < 0.05. ** P < 0.01. Ad-NC, cells infected with empty adenovirus; Ad-HSPA12A, cells infected with HSPA12A-adenovirus; HG-CD147, high-glycosylation CD147; NoG-CD147, non-glycosylation CD147; HRD1, HMG-CoA reductase degradation protein 1.

Considering that ubiquitination is necessary for proteasomal protein degradation, we examined the effects of HSPA12A on CD147 ubiquitination. To this end, ubiquitin-conjugated proteins from HSPA12A-overexpressing (Ad-HSPA12A) Caki-1 cells and the control (Ad-NC) cells were immunoprecipitated with the antibody against ubiquitin. The ubiquitin-precipitates from Ad-HSPA12A cells showed higher HG-CD147 protein content than the precipitates from Ad-NC cells and a higher level of non-glycosylated CD147 (NoG-CD147) was recovered rather than LG-CD147 (**Figure 9A**). Conversely, HSPA12A deficiency significantly reduced the ubiquitination of both HG-CD147 and NoG-CD147 compared with that in control cells using immunoprecipitation immunoblotting analyses (**Figure S18**).

### HSPA12A interacts with HMG-CoA reductase degradation protein 1 (HRD1), an E3 ubiquitin ligase

HRD1 is a ubiquitin E3 ligase that ubiquitinates and degrades CD147 44, 45. To examine whether HRD1 was involved in the HSPA12A-induced CD147 proteasomal degradation, we first evaluated the effects of HSPA12A on HRD1 expression. A significant increase of HRD1 protein expression was found in both Caki-1 and 786O cells following HSPA12A overexpression compared to their Ad-NC controls (**Figure 9B**). Notably, immunoprecipitation-immunoblotting analysis revealed the presence of HRD1 protein in the Flag-tagged HSPA12A immuno-precipitates from Ad-HSPA12A Caki-1 or 786O cells, showing the interaction of HSPA12A with HRD1 in RCC cells (**Figure 9C**).

## Discussion

In this study, we identified HSPA12A as a novel negative regulator of renal cancer cell migration and metastasis. HSPA12A's function, as a potential migration- and metastasis suppressor, was mediated by modulating CD147 stability and CD147-mediated lactate export and glycolysis (**Figure 9D**). These findings indicate that promoting HSPA12A expression could be an effective strategy for the management of RCC metastasis.

HSPA12A belongs to the evolutionarily conserved superfamily of HSPs, comprising a group of structurally unrelated subfamilies, including HSPA/HSP70, HSPB/HSP27, HSPC/HSP90, HSPH/HSP110, and NDAJ/HSP40 46. Several HSPs, especially HSP27, HSP72, and HSP90, are upregulated in a variety of human cancers (e.g. hepatocellular and prostate carcinomas) to form a fostering environment essential for tumor growth, invasion, and metastasis, leading to secondary cancers by chaperoning uncontrolled proliferation, angiogenesis, epithelial-mesenchymal transition, and escaping cell death and senescence 47. In renal cancers, HSP27, HSP72, and HSP90 show a heterogeneous expression pattern. However, HSP40 family protein DNAJB8 is upregulated in renal cancers and induces tumorigenesis by maintaining cancer stem-like cells, a population of cancer cells with tumor initiation, self-renewal, and differentiation properties 48, 49.

In the present study, we detected a downregulated expression of HSPA12A in human RCC tumors, which was associated with advanced TNM stage and Fuhrman grade, as well as larger tumor size. The Kaplan-Meier plotter from the GEPIA database showed a correlation between low *Hspa12a* mRNA expression and poor survival in RCC patients. Loss- and gain-of HSPA12A function experiments in human RCC cells showed that HSPA12A did not directly regulate RCC cell proliferation but exhibited a negative correlation with RCC cell migration. We also found that HSPA12A negatively regulated phosphorylation of FAK as well as the expression of integrin-β1 and MMP2/9. FAK is a multifunctional regulator of cell signaling within the tumor microenvironment and is at the intersection of various signaling pathways that promote cancer metastasis. It can be activated by integrin-β to increase the expression or activation of MMPs, which, in turn, facilitate tumor cell invasion into the surrounding microenvironment 31-33. Various other tumor-promoting signaling pathways are also involved in FAK's function. Thus, FAK inhibitors are emerging as promising chemotherapeutics for tumor metastasis in mouse models. In our study, inhibition of FAK diminished the HSPA12A knockdown-induced promotion of RCC cell migration, suggesting that FAK signaling was involved in the regulation of HSPA12A in RCC migration. These findings suggest that the downregulation of HSPA12A expression was associated with poor prognosis in human RCC, which could be attributed to the negative regulatory effect of HSPA12A on RCC cell migration. The results provided insights into the role of heat shock proteins in cancer progression.

CD147 is a glycoprotein that was initially identified as a regulator of MMPs. Increasing evidence indicates that CD147 is overexpressed in cancer cells and involved in promoting cancer cell metastasis through several mechanisms. One important mechanism underlying the effect of CD147 on promoting cancer metastasis is the metabolic modification of the tumor microenvironment through interactions with specific MCTs, such as MCT4, to facilitate lactate export and tumor glycolysis 37, 50. Moreover, CD147 induces MMPs expression by activating FAK in an integrin-dependent manner 50. However, little is known about the regulation of heat shock proteins in CD147 expression. Here, we found that human RCC tumors showed reduced HSPA12A accompanied by increased CD147 protein expression, suggesting that downregulation of HSPA12A might increase RCC cell migration by upregulating CD147. This hypothesis was supported by the findings that HSPA12A negatively regulated CD147 protein abundance and membrane expression in RCC cells. Moreover, HSPA12A negatively regulated lactate export, which was not caused by changes in lactate generation. In view of previous reports showing that intracellular lactate accumulation serves as a negative feedback signal for glycolysis 9, 20, the present data suggest that the downregulation of HSPA12A might increase lactate export to upregulate glycolysis. Indeed, the glycolysis rate, glycolytic capacity and expression of glycolysis-related genes were promoted by HSPA12A knockdown in RCC cells. More importantly, overexpression of CD147 reversed the HSPA12A overexpression-induced inhibition of RCC cell migration, lactate export, and the glycolysis-related gene expression. These data suggest that the downregulation of HSPA12A in RCC cells increases migration and glycolysis in a CD147-dependent manner.

Previous studies have reported that CD147 can promote the proliferation of some cancer cells such as hepatocellular carcinoma cells through multiple pathways such as PI3K/Akt signaling 51, 52. Though we found a negative regulation of HSPA12A in CD147 protein abundance, the proliferation of both Caki-1 and 786O RCC cells was not affected by knockdown or overexpression of HSPA12A. The Akt activation in RCC cells was also not affected by HSPA12A (**Figure S19**). When taken into account that our recent reports have shown a promoted proliferation of hepatocellular carcinoma cells by HSPA12A 53, the current data suggest that the effects of CD147 and HSPA12A on proliferation are cancer cell type- and signaling activation-dependent.

Evidence has shown that CD147 is degraded through the ubiquitin-proteasome system, and HRD1 is an E3 ligase involved in CD147 ubiquitination 54. Previous studies have demonstrated that HRD1 is an endoplasmic reticulum-associated ubiquitin ligase involved in CD147 degradation in human hepatocellular carcinoma cells. Moreover, HRD1 has been shown to form a signaling axis with HSP70 to regulate the onco-repressor potential of N-terminal misfolded Blimp-1s in lymphoma cells 55. To understand the increased CD147 protein expression by downregulated HSPA12A, we analyzed its effect on *Cd147* mRNA levels and found that HSPA12A did not affect *Cd147* mRNA expression in RCC cells. Our results were supported by the TCGA database which showed a comparable *Cd147* mRNA expression between RCC patients and the normal controls. We speculated that HSPA12A might regulate CD147 protein abundance by modulating its stability. The following findings supported this hypothesis. (1) Protein synthesis blocker CHX caused a gradual decrease in CD147 protein abundance in control RCC cells; however, the CHX-induced reduction of CD147 protein abundance was attenuated by HSPA12A knockdown, (2) Treatment with the proteasome inhibitor MG132 abolished the HSPA12A overexpression-induced decrease of CD147 protein, (3) In the ubiquitin immuno-precipitates from HSPA12A-overexpressing RCC cells, CD147 was present at a higher level, and the opposite pattern was observed in response to HSPA12A knockdown, and (4) HSPA12A showed an interaction with E3 ubiquitin ligase HRD1. These results suggest that HSPA12A negatively regulates CD147 protein stability by modulating CD147 ubiquitination for proteasomal degradation.

## Conclusion

Our study demonstrated that the loss of HSPA12A was associated with metastasis in human RCC, while overexpression of HSPA12A inhibited RCC cell migration. We further provided evidence that the anti-metastatic action of HSPA12A was caused by the negative regulation of CD147 stability and the CD147-mediated lactate export (**Figure 9D**). The present data suggest that increasing HSPA12A expression provides an effective strategy for preventing migration of human RCC cells.

## Supplementary Material

Supplementary figures and tables.

## Abbreviations

Si-NC: Scramble-transfected controls
Si-HSPA12A: HSPA12A Knockdown by HSPA12A siRNA
Ad-NC: empty adenovirus-infected controls
Ad-HSPA12A: HSPA12A overexpression by infection with HSPA12A adenovirus
FAK: Focal adhesion kinase
MMP: Matrix metallopeptidase
ERK: Extracellular signal-regulated kinase
GLUT1: Glucose transporter 1
GLUT4: Glucose transporter type 4
HK2: Hexokinase 2
PFKFB3: 6-phosphofructo-2-kinase/fructose-2:6-biphosphatase 3
LDHA: Lactate dehydrogenase A
MCT4: Monocarboxylate transporter 4
HG-CD147: high-glycosylation CD147
LG-CD147: low-glycosylation CD147
CHX: cycloheximide
HRD1: HMG-CoA reductase degradation protein 1
