# Supplementary material for: HSPA12A unstabilizes CD147 to inhibit lactate export and migration in human renal cell carcinoma
Source: Theranostics. 2020 Jul 9;10(19):8573–90. doi: 10.7150/thno.44321 (PMC7392002; doi:10.7150/thno.44321)
Supplement: Supplementary file 1 — Supplementary figures and tables. [file thnov10p8573s1.pdf]

Supplementary tables

Table S1. SiRNA sequence used in the experiments

Species	Gene name		Sequence (5'-3')
human	*Hspa12a*	Sense	GGAGAAUGUCAUAGGAGAATT
		Antisense	UUCUCC UAUGACAUUCUCCTT

Table S2. Lysis Buffer used in cytosolic and pellet protein extracts

Lysis Buffer A (10ml) for cytosolic protein

	Storage Concentration	Working Concentration	Volume(ml)
Tris (HCl, pH 7.4)	1 M	5 mM	0.05
EDTA (pH 8.0)	250 mM	5 mM	0.1
EGTA (pH 8.0)	250 mM	10 mM	0.4
NaCl	3 M	150 mM	0.5
PI	25×		0.4
PPI	10×		2
PNPP	500 mM	20 mM	0.4
DTT	400 mM	1 mM	0.025
H2O			6.125

Lysis Buffer B (4ml) for pellet protein

NP-40(10%)	0.2 ml
Brij-35(10%)	0.08 ml
Sodium deoxycholate	0.08 ml
DTT	0.01 ml
LysisA	3.63 ml

Table S3. Antibodies used in the experiments

Antibody	Source	Company	Catalog No.
Anti-HSPA12A	Rabbit	Abcam	ab200838
Anti-Lamin A/C	Rabbit	ProteinTech	10298-1-AP
Anti-Bax	Rabbit	Cell Signaling	#2772
Anti-Bcl2	Rabbit	Bioworld Technology	BS1511
Anti-C-Myc	Rabbit	ProteinTech	10828-1-AP
Anti- Cyclin D1	Mouse	ProteinTech	60186-1-Ig
Anti-Integrinβ1	Rabbit	Cell Signaling	#9699
Anti-p-FAK	Rabbit	Bioworld Technology	BS4718
Anti-FAK	Rabbit	Bioworld Technology	BS3581
Anti-p-ERKs	Rabbit	Cell Signaling	#9101
Anti-ERKs	Rabbit	Cell Signaling	#9102
Anti-MMP2	Rabbit	Bioworld Technology	BS1236
Anti-MMP7	Rabbit	ProteinTech	10374-2-AP
Anti-MMP9	Rabbit	Bioworld Technology	BS6893
Anti-CD147	Rabbit	ProteinTech	11989-1-AP
Anti-MCT4	Rabbit	ProteinTech	22787-1-AP
Anti-GLUT1	Rabbit	Cell Signaling	#12939S
Anti-GLUT4	Rabbit	Abcam	ab33780
Anti-PFKFB3	Rabbit	Cell Signaling	#13123S
Anti-LDHA	Rabbit	Abcam	ab101562
Anti-Flag	Mouse	Sigma-Aldrich	F1804
Anti-IgG	Mouse	SantaCruz	sc-2025
Anti-Ubiquitin (F-11)	Mouse	SantaCruz	sc-271289
Anti-HRD1 (SYVN1)	Rabbit	Bioworld Technology	BS70501
Anti-GAPDH	Rabbit	Bioworld Technology	AP0063
Anti-HUMAN CD147	Mouse	eBioscience	17-1472-42

IgG1 kappa Isotype Control	Mouse	eBioscience	17-4714-81
Anti-p-Akt	Rabbit	Cell Signaling	#9271

Table S4. Primers used in the experiments

Genes	Primer Sequences	
Cd147	Forward	ACTCCTCACCTGCTCCTTGA
	Reverse	GCCTCCATGTTCAGGTTCTC
Bcl2	Forward	GGTGGGGTCATGTGTGTGG
	Reverse	CGGTTCAGGTACTCAGTCATCC
Bax	Forward	CCCGAGAGGTCTTTTTCCGAG
	Reverse	CCAGCCCATGATGGTTCTGAT
Cyclin d1	Forward	GCTGCGAAGTGGAAACCATC
	Reverse	CCTCCTTCTGCACACATTTGAA
C-Myc	Forward	GGCTCCTGGCAAAAGGTCA
	Reverse	CTGCGTAGTTGTGCTGATGT
Mct4	Forward	AGGTATCCTTGAGACGGTCAG
	Reverse	CAAGCAGGTTAGTGATGCCG
Gapdh	Forward	ACAACTTTGGTATCGTGGAAGG
	Reverse	GCCATCACGCCACAGTTTC

A

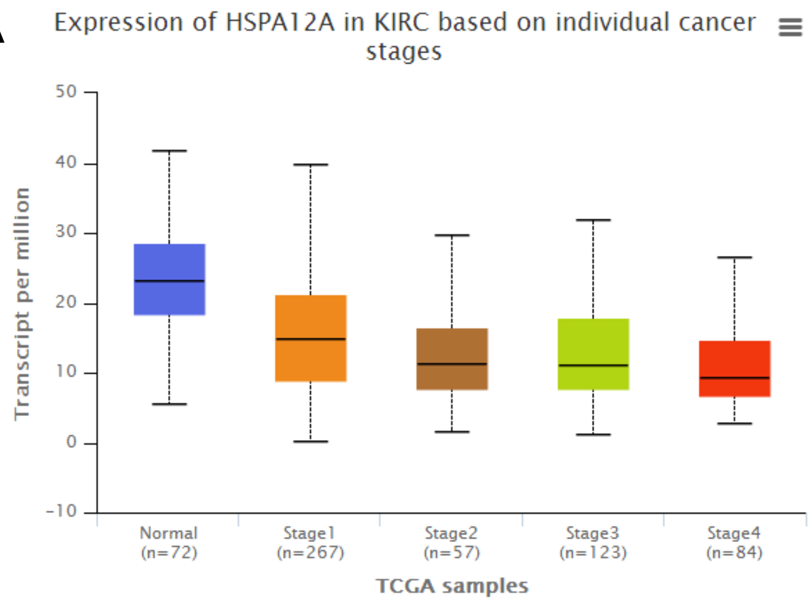

Comparision	Statistical significance
Normal-vs-Stage1	1.79439796355041E-11
Normal-vs-Stage2	5.41344746807226E-13
Normal-vs-Stage3	4.20440349202522E-11
Normal-vs-Stage4	1.72506453566257E-12
Stage1-vs-Stage2	2.024400E-02
Stage1-vs-Stage3	1.136580E-01
Stage1-vs-Stage4	3.881700E-03
Stage2-vs-Stage3	4.162000E-01
Stage2-vs-Stage4	8.362000E-01
Stage3-vs-Stage4	3.055000E-01

B

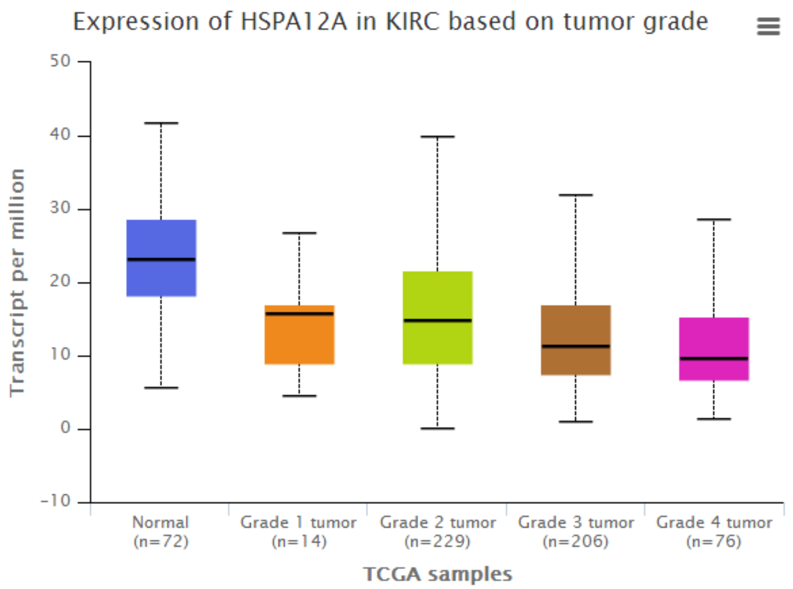

Comparision	Statistical significance
Normal-vs-Grade1	3.593500E-04
Normal-vs-Grade2	9.30600041471052E-12
Normal-vs-Grade3	1.62481139653892E-12
Normal-vs-Grade4	1.80422343731834E-12
Grade1-vs-Grade2	9.155000E-01
Grade1-vs-Grade3	4.828800E-01
Grade1-vs-Grade4	2.695800E-01
Grade2-vs-Grade3	2.281700E-02
Grade2-vs-Grade4	1.110170E-02
Grade3-vs-Grade4	3.659600E-01

Figure S1. Downregulated HSPA12A was related with advanced human RCC progression.

(A) HSPA12A mRNA levels were significantly down-regulated in all KIRC stages as compared to non-tumor kidney tissues in TCGA database from UALCAN website. The statistical significance and sample numbers are shown in Figures. (B) HSPA12A mRNA levels were significantly down-regulated in all KIRC grades as compared to non-tumor kidney tissues in TCGA database from UALCAN website. The statistical significance and sample numbers are shown in Figures. TCGA-KIRC, the Cancer Genome Atlas database for Kidney Renal Clear Cell Carcinoma.

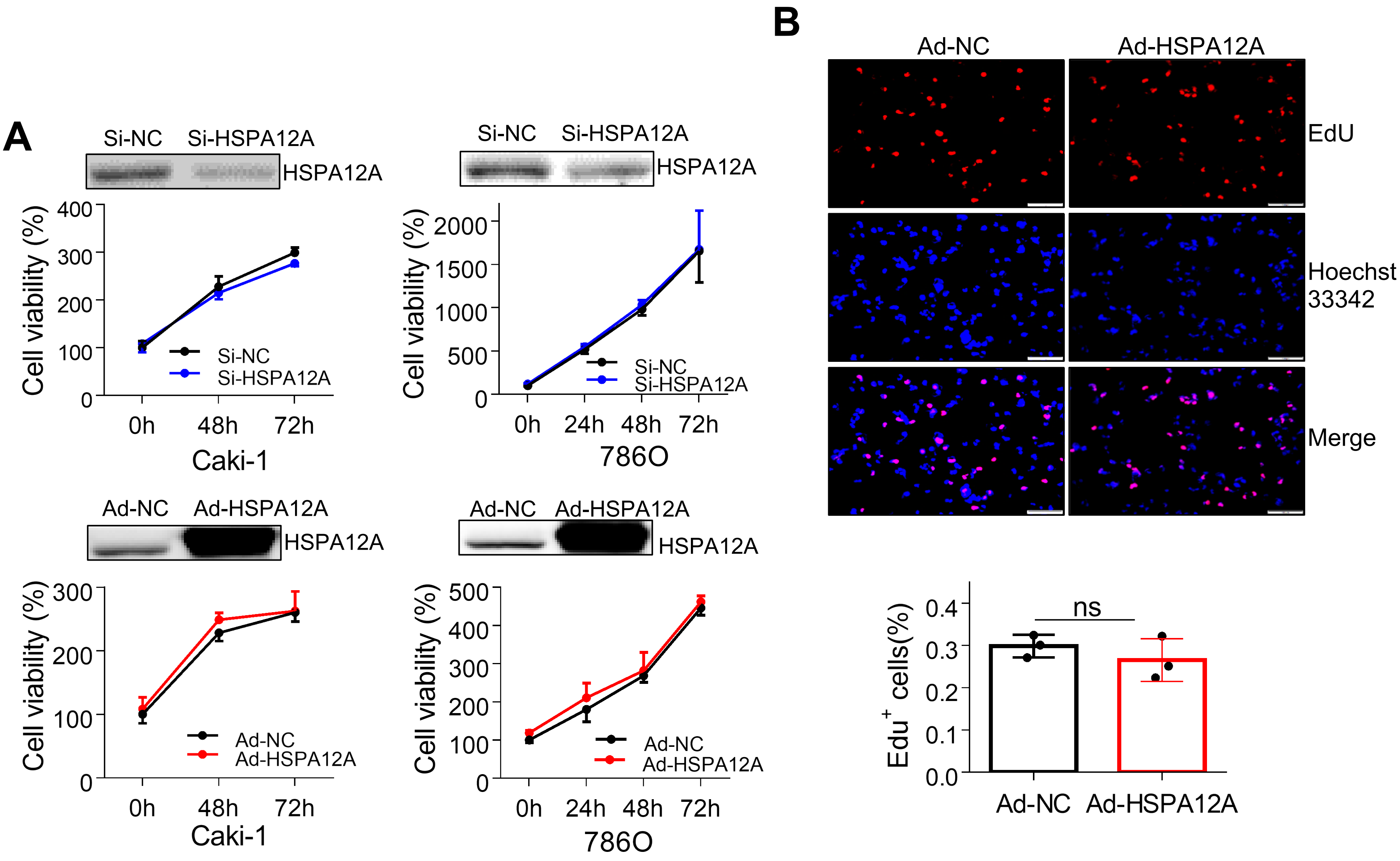

Figure S2. HSPA12A showed no effects on RCC cell proliferation.

(A) Viability in Caki-1 and 786O cells was estimated by MTT assay after overexpression or knockdown of HSPA12A for the indicated times. The viability was expressed as the percentage over their corresponding NC controls at 0 h. n = 6/group. (B) Following overexpression of HSPA12A for 46 h, Caki-1 cells were incubated with EdU for another 2 h. Cell proliferation was indicated by EdU incorporation assay. Scale bar = 100 μm; ns, no statistical significance; n = 3/group. Si-NC, cells transfected with Scramble siRNA; Si-HSPA12A, cells transfected with HSPA12A-targeted siRNA; Ad-NC, cells infected with empty adenovirus; Ad-HSPA12A, cells infected with HSPA12A-adenovirus.

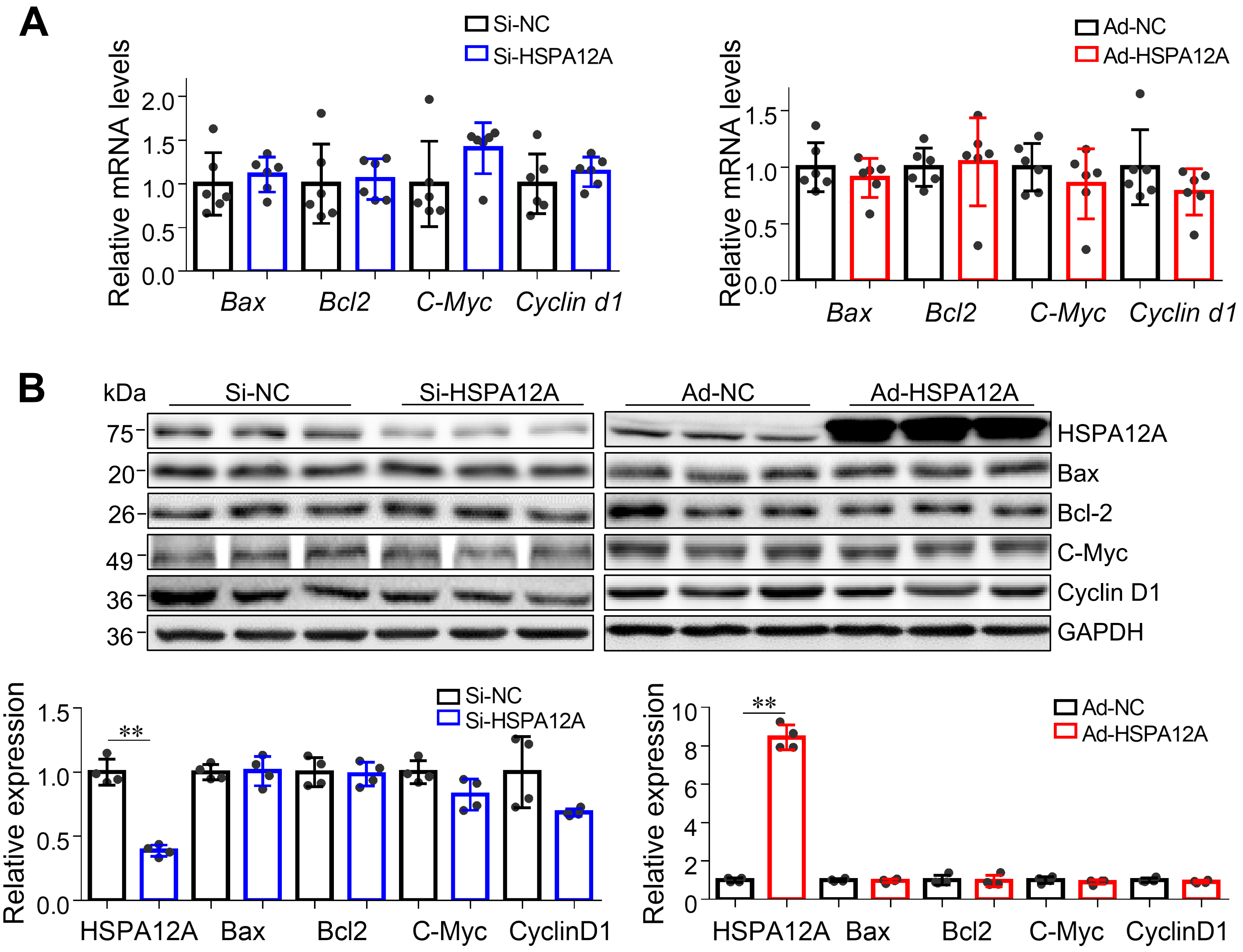

Figure S3. HSPA12A showed no effects on the expression of genes that associated with proliferation and survival in RCC cells. Following overexpression or knockdown of HSPA12A for 48 h, Caki-1 cells were collected for quantitative PCR (A) and immunoblotting (B) analyses for the indicated genes. n = 6/group (A), n = 4/group (B). ** *P* < 0.01. Si-NC, cells transfected with Scramble siRNA; Si-HSPA12A, cells transfected with HSPA12A-targeted siRNA; Ad-NC, cells infected with empty adenovirus; Ad-HSPA12A, cells infected with HSPA12A-adenovirus.

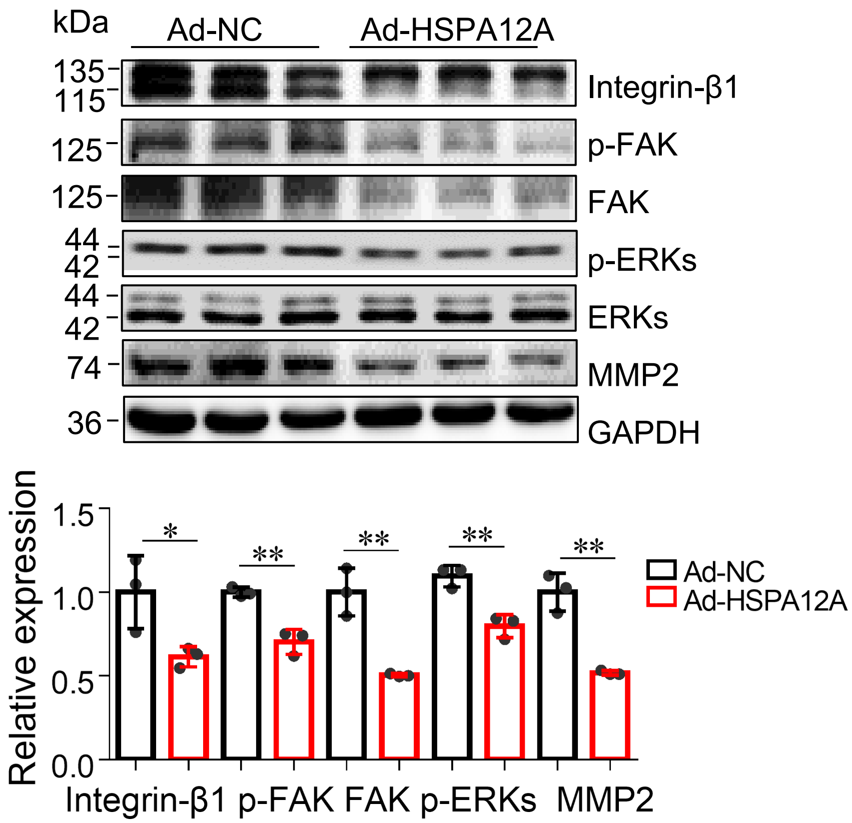

Figure S4. HSPA12A reduced expression of genes related to migration in RCC 786O cells.

Immunoblotting was performed against the indicated proteins in 786O cells following overexpression of HSPA12A for 48 h. n = 3/group. * *P* < 0.05, ** *P* < 0.01. Ad-NC, cells infected with empty adenovirus; Ad-HSPA12A, cells infected with HSPA12A-adenovirus.

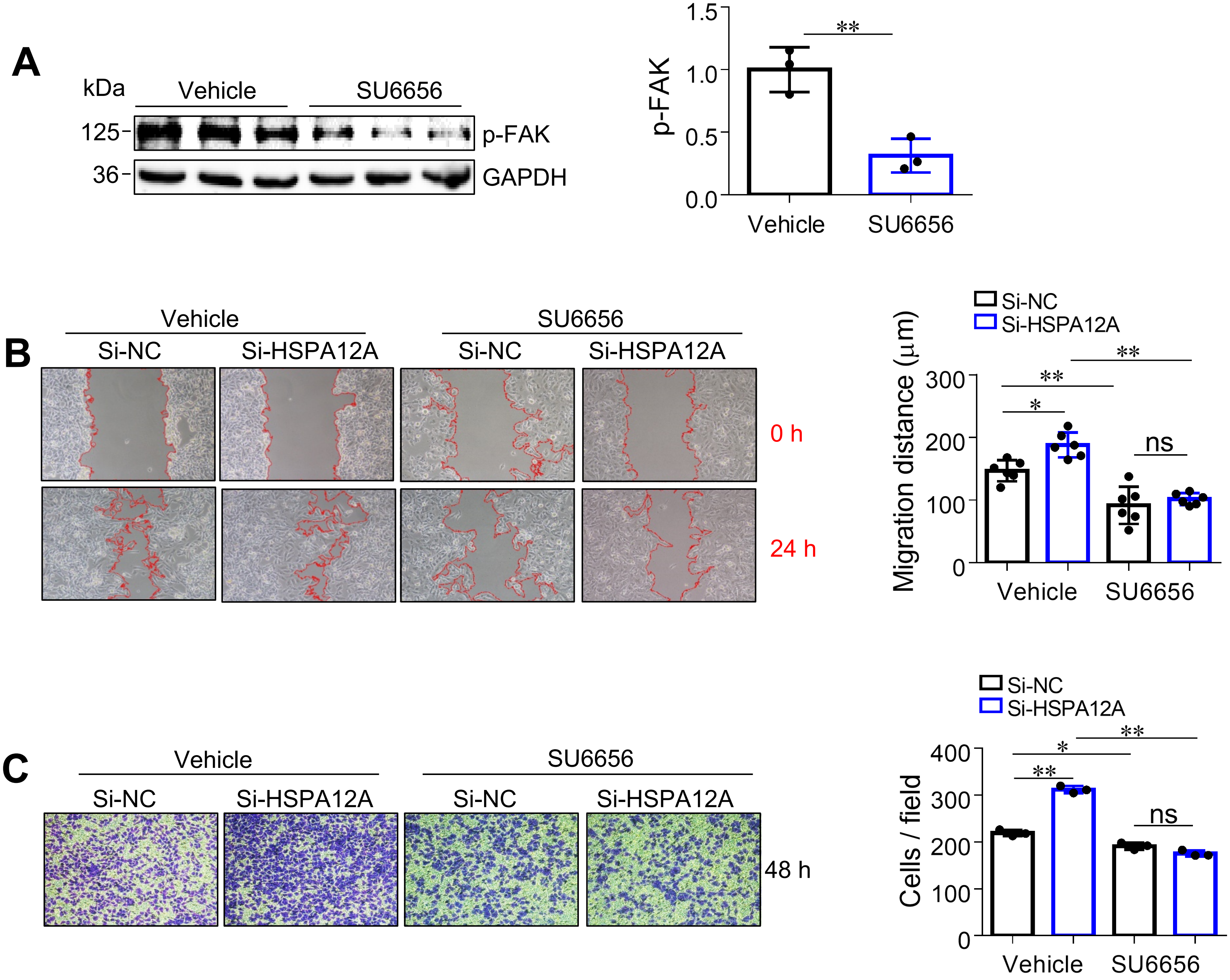

Figure S5. Inhibition of FAK diminished the HSPA12A knockdown-induced promotion of RCC cell migration.

(A) Inhibition of FAK phosphorylation by SU6656 in Caki-1 cells. (B) The wound was made in Caki-1 cell layers after knockdown of HSPA12A for 48 h in the presence or absence of SU6656, and the migration distance was measured at 24 h after wounding. (C) The extent of cell migration was assessed in Caki-1 cells after knockdown of HSPA12A for 48 h by Transwell migration assay in the presence or absence of SU6656. The migrated cells stained with crystal violet were observed 48 h later. n = 3/group (A, C) and n = 6/group (B). * $P < 0.05$, ** $P < 0.01$. Si-NC, cells transfected with Scramble siRNA; Si-HSPA12A, cells transfected with HSPA12A-targeted siRNA; ns, no significance.

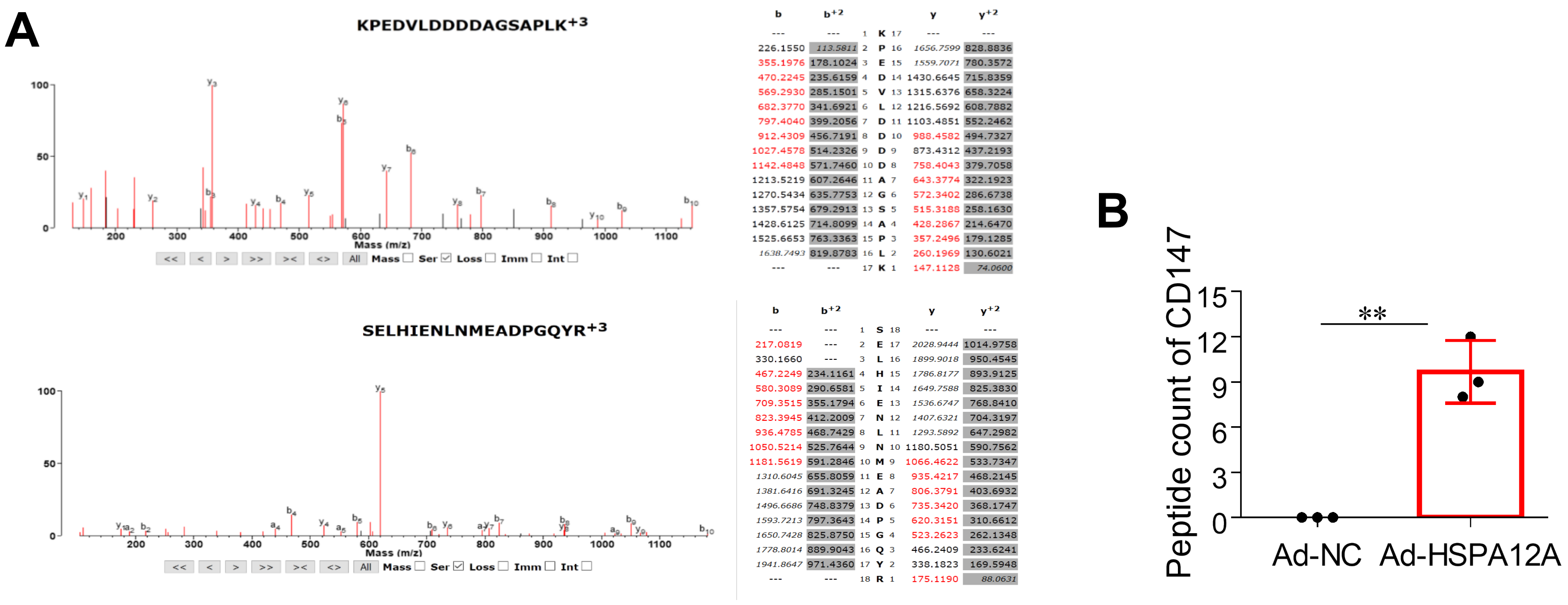

Figure S6. Mass spectrum indicated an interaction of HSPA12A with CD147 in hepatocellular carcinoma HepG2 cells.

(A) MS/MS and main sequence ions of a representative CD147 peptide. (B) Comparison of peptide count of CD147 identified in HSPA12A immunoprecipitated from Ad-NC and Ad-HSPA12A HepG2 cells. Note that the Ad-HSPA12A HepG2 group has an average of 9.67 peptide count, compared to 0 peptide count in the Ad-NC-group, indicating a significant increase of CD147-HSPA12A in hepatocellular carcinoma HepG2 cells. n = 3/group. ** $P < 0.01$. Ad-NC, cells infected with empty adenovirus; Ad-HSPA12A, cells infected with HSPA12A-adenovirus.

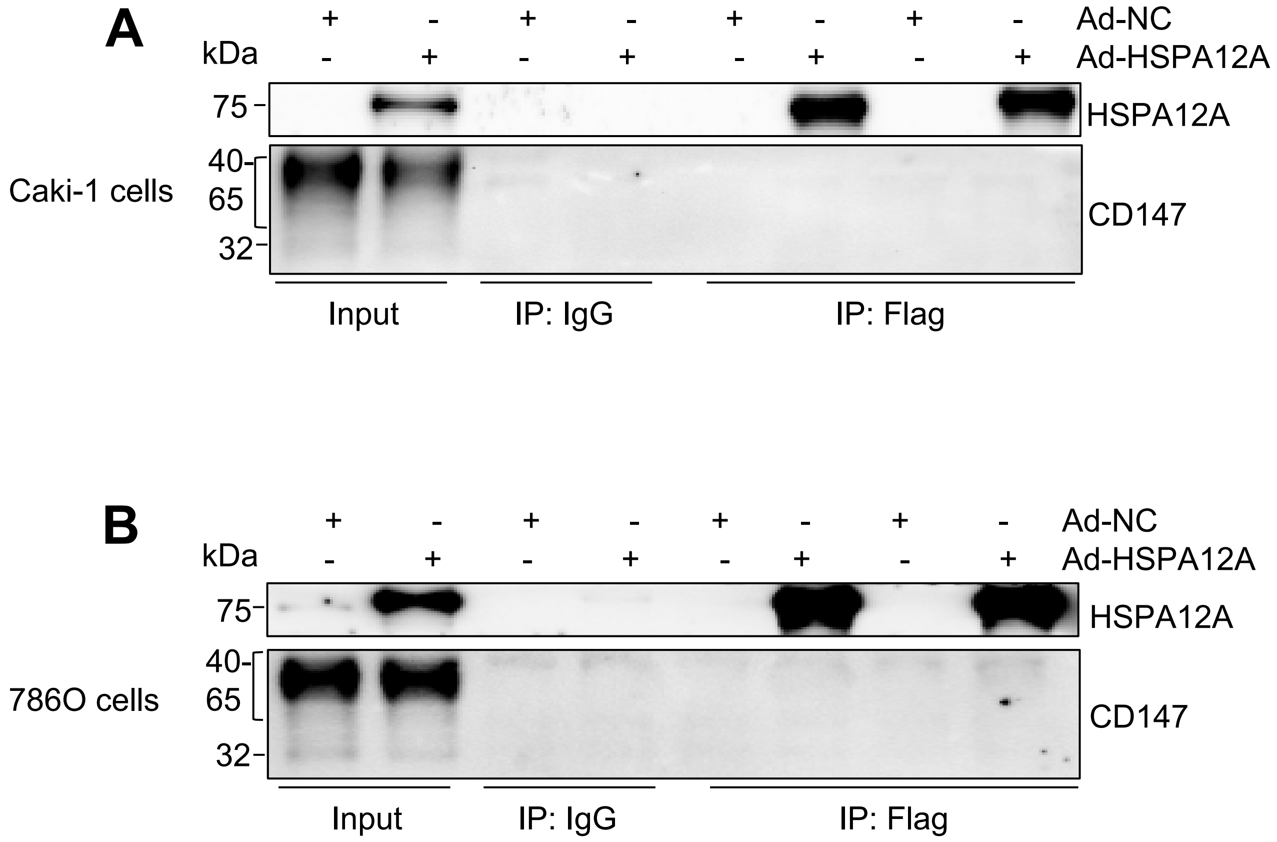

Figure S7. HSPA12A had no direct interaction with CD147 in RCC Caki-1 and 786O cells.

Cellular protein extracts from Ad-NC and Ad-HSPA12A Caki-1 (A) or 786O (B) cells were immunoprecipitated with anti-Flag. The immunocomplexes were immunoblotted with HSPA12A and CD147 respectively. Protein extracts without immunoprecipitation (input) served as positive controls, and immunoprecipitates from IgG incubation served as negative controls. Ad-NC, cells infected with empty adenovirus; Ad-HSPA12A, cells infected with HSPA12A-adenovirus.

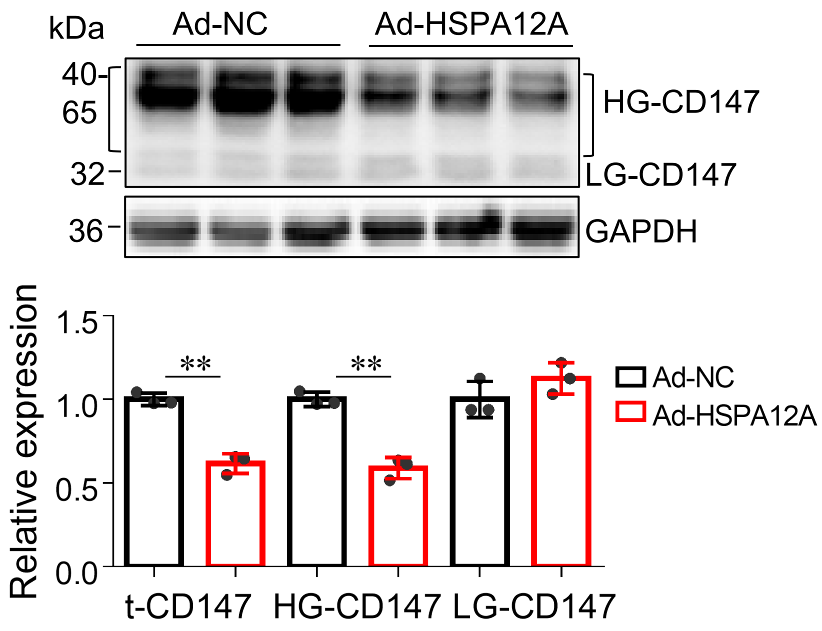

Figure S8. Effects of HSPA12A on CD147 expression in RCC 786O cells.

Immunoblotting was performed in 786O cells following HSPA12A overexpression for 48 h. n = 3/group. ** *P* < 0.01. Ad-NC, cells infected with empty adenovirus; Ad-HSPA12A, cells infected with HSPA12A-adenovirus; t-CD147, total CD147; HG-CD147, high-glycosylation CD147; LG-CD147, low-glycosylation CD147.

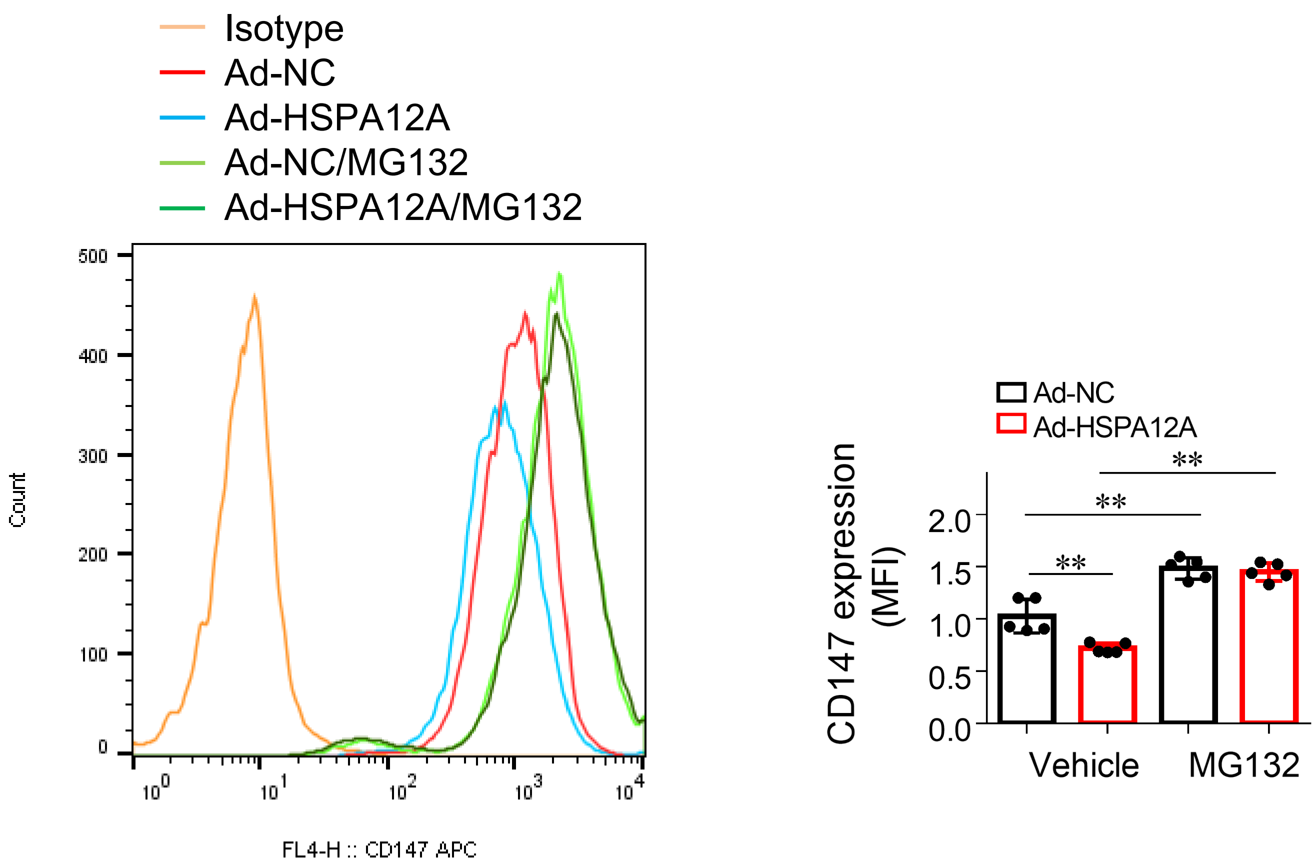

Figure S9. Flow cytometry analysis.

Expression of CD147 was estimated by flow cytometry after overexpression of HSPA12A for 48 h in the presence or absence of MG132. The mean fluorescence intensity (MFI) of CD147 was presented as relative levels over the vehicle-treated Ad-NC controls. n = 5/group. ** $P < 0.01$. Ad-NC, cells infected with empty adenovirus; Ad-HSPA12A, cells infected with HSPA12A-adenovirus.

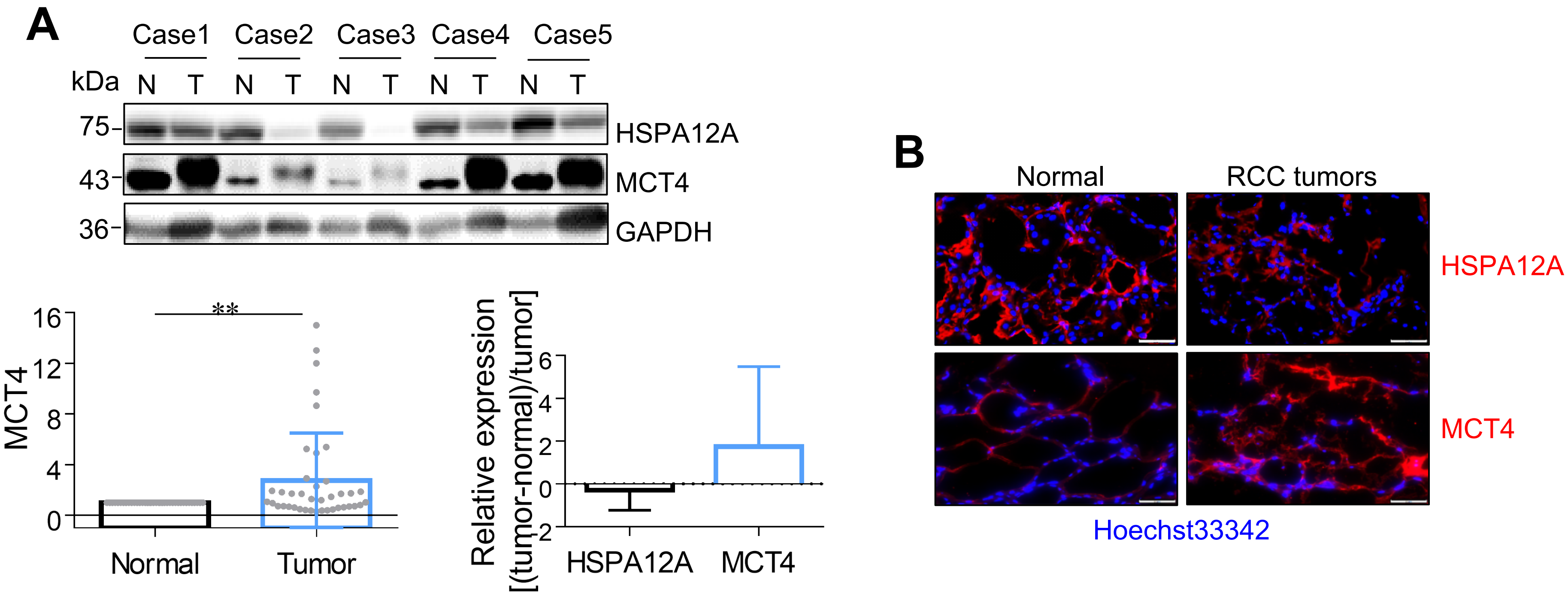

Figure S10. MCT4 expression in RCC tumors.

(A) Immunoblotting was performed in human RCC tumors and the matched non-tumor kidney tissues. n = 40/group. ** *P* < 0.01. (B) Immunofluorescence staining was performed on frozen sections of human RCC tumors and the matched non-tumor kidney tissues. Hoechst 33342 was used to counterstain the nuclei. Scale bar = 50 μm; n = 3 human subjects/group. N, normal kidney tissues; T, RCC tumors.

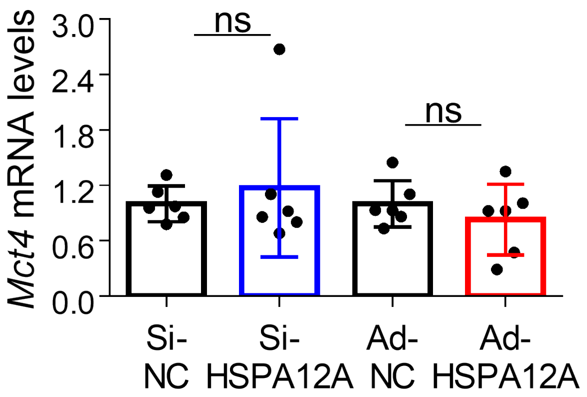

Figure S11. Effects of HSPA12A on *Mct4* mRNA expression in Caki-1 cells.

Levels of *Mct4* mRNA were examined in Caki-1 cells following HSPA12A knockdown or overexpression for 48 h. n = 6/group. ns, no significance. Si-NC, cells transfected with Scramble siRNA; Si-HSPA12A, cells transfected with HSPA12A-targeted siRNA; Ad-NC, cells infected with empty adenovirus; Ad-HSPA12A, cells infected with HSPA12A-adenovirus.

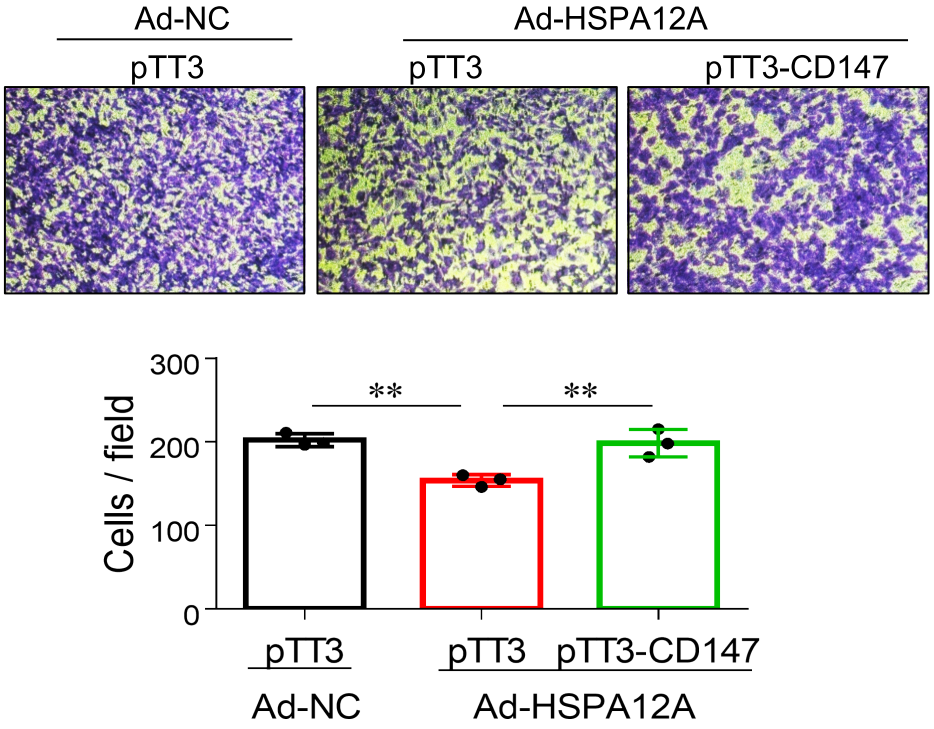

Figure S12. Overexpression of CD147 reversed the HSPA12A-induced inhibition of migration in RCC 786O cells.

Twenty-four hours after HSPA12A overexpression, 786O cells (Ad-HSPA12A) were transfected with pTT3-CD147 plasmids to overexpress CD147 or transfected with pTT3 control plasmids. The extent of cell migration was assessed by Transwell migration assay 48 h later. n = 3/group. ** *P* < 0.01. Ad-NC, cells infected with empty adenovirus; Ad-HSPA12A, cells infected with HSPA12A-adenovirus; pTT3, empty pTT3 plasmid; pTT3-CD147, plasmid expressing CD147.

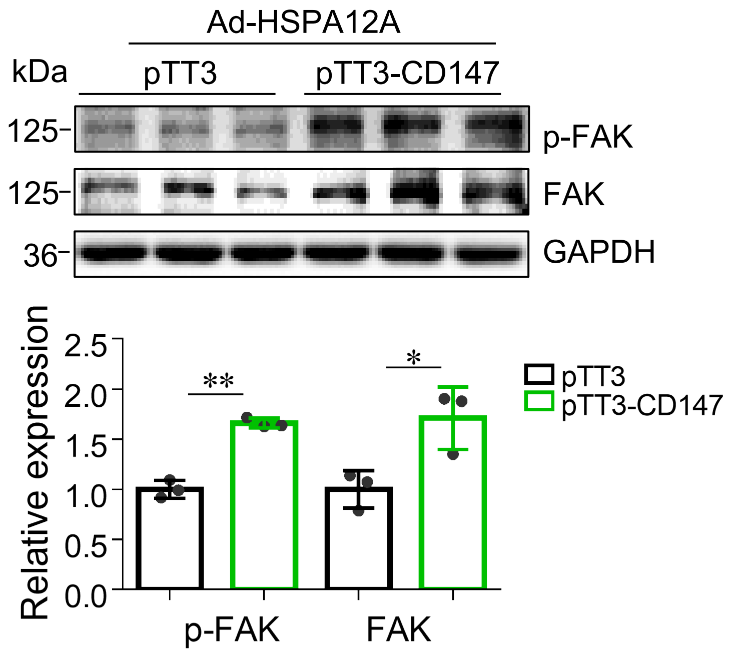

Figure S13. Overexpression of CD147 increased FAK phosphorylation in HSPA12A overexpressing-786O cells.

Twenty-four hours after HSPA12A overexpression, 786O cells (Ad-HSPA12A) were transfected with pTT3-CD147 plasmids to overexpress CD147 or transfected with pTT3 control plasmids. The immunoblotting analysis was performed 48 h after plasmid transfection. n = 3/group. * $P < 0.05$, ** $P < 0.01$. Ad-HSPA12A, cells infected with HSPA12A-adenovirus; pTT3, empty pTT3 plasmid; pTT3-CD147, plasmid expressing CD147.

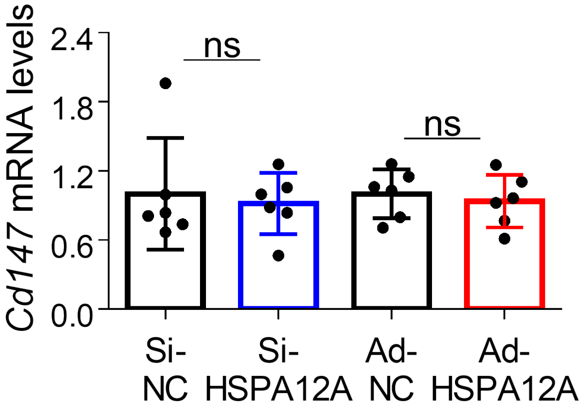

Figure S14. Effects of HSPA12A on *Cd147* mRNA expression in 786O cells.

Levels of *Cd147* mRNA were examined in 786O cells following HSPA12A knockdown or overexpression for 48 h. n = 6/group. ns, no significance. Si-NC, cells transfected with Scramble siRNA; Si-HSPA12A, cells transfected with HSPA12A-targeted siRNA; Ad-NC, cells infected with empty adenovirus; Ad-HSPA12A, cells infected with HSPA12A-adenovirus.

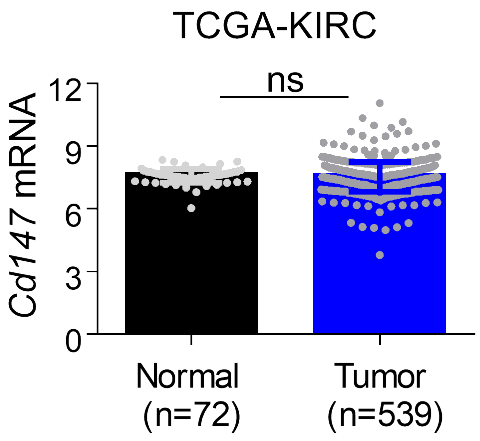

Figure S15. *Cd147* mRNA expression in human RCC tumors.

Cd147 mRNA levels were obtained from TCGA-KIRC database. Sample numbers were indicated in figure. TCGA-KIRC, the Cancer Genome Atlas database for Kidney Renal Clear Cell Carcinoma. ns, no significance.

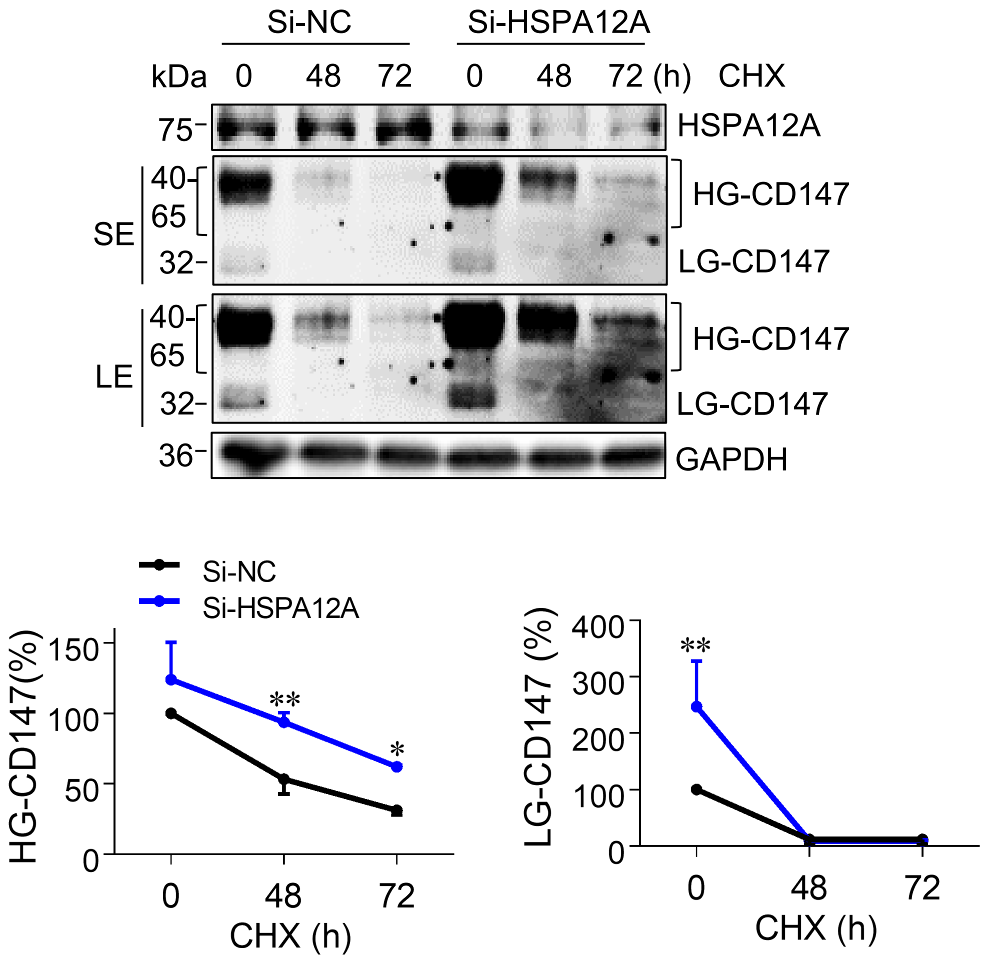

Figure S16. HSPA12A negatively regulated CD147 stability in 786O cells.

Twenty-four hours after HSPA12A knockdown, 786O cells were treated with cycloheximide (CHX, 150 μg/ml) for the indicated durations. CD147 protein abundance was examined by immunoblotting and expressed as the percentage of the NC contents at 0 h. n = 3/group, * $P < 0.05$, ** $P < 0.01$ vs. the time matched Si-NC group. Si-NC, cells transfected with Scramble siRNA; Si-HSPA12A, cells transfected with HSPA12A-targeted siRNA; SE, short exposure; LE, long exposure; HG-CD147, high-glycosylation CD147; LG-CD147, low-glycosylation CD147; CHX, cycloheximide.

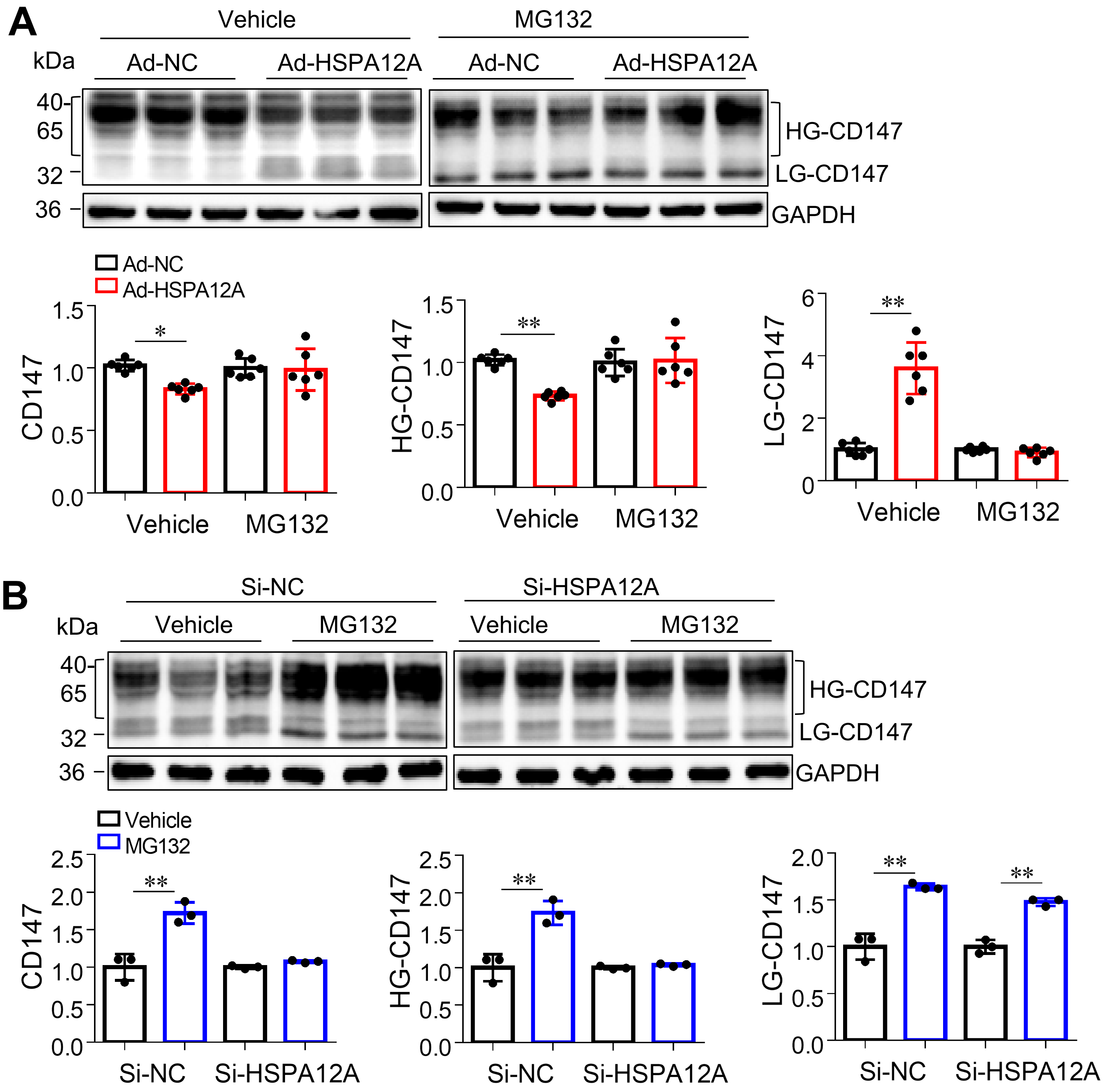

Figure S17. Effects of proteasome inhibitor MG132 on HSPA12A-induced decrease of CD147 in 786O cells.

Forty-eight hours after HSPA12A overexpression (A) or knockdown (B), 786O cells were treated with MG132 (20 μM) for 8 h. CD147 protein abundance was examined by immunoblotting. n = 6/group (A) and n = 3/group (B). * $P < 0.05$, ** $P < 0.01$. Si-NC, cells transfected with Scramble siRNA; Si-HSPA12A, cells transfected with HSPA12A-targeted siRNA; Ad-NC, cells infected with empty adenovirus; Ad-HSPA12A, cells infected with HSPA12A-adenovirus; HG-CD147, high-glycosylation CD147; LG-CD147, low-glycosylation CD147.

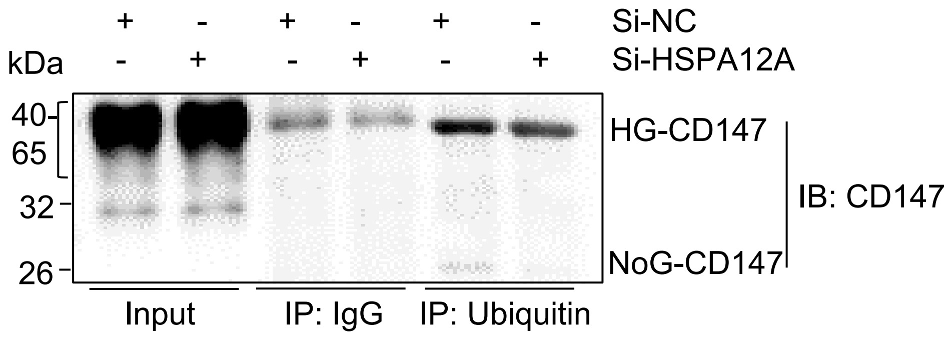

Figure S18. HSPA12A knockdown reduced CD147 ubiquitination.

Forty-eight hours after HSPA12A knockdown, Caki-1 cells were collected. Cellular protein extracts were immunoprecipitated with primary antibody for ubiquitin. The immunoprecipitates were blotted with CD147. Protein extracts without immunoprecipitation (input) served as positive controls, and immunoprecipitates from IgG incubation served as negative controls. Si-NC, cells transfected with Scramble siRNA; Si-HSPA12A, cells transfected with HSPA12A-targeted siRNA; HG-CD147, high-glycosylation CD147; NoG-CD147, non-glycosylation CD147.

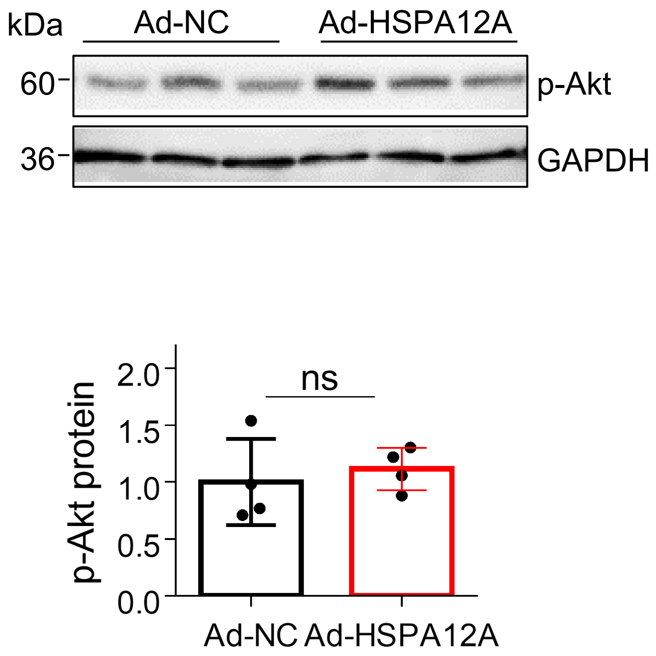

Figure S19. HSPA12A showed no effect on Akt phosphorylation protein levels.

Immunoblotting was performed in Caki-1 cells following HSPA12A overexpression for 48 h. n = 4/group. ns, no significance; Ad-NC, cells infected with empty adenovirus; Ad-HSPA12A, cells infected with HSPA12A-adenovirus.
